# Garciniagifolone A, derived from *Garcinia multiflora* fruits, inhibits triple-negative breast cancer cells and organoids growth by targeting CA9

**DOI:** 10.3389/fcell.2026.1767397

**Published:** 2026-03-11

**Authors:** Fulin Zhou, Siyu Yang, Qiang Lin, Ruling Zhang, Yingshi Su, Yibo Hou, Jin Wang, Jiaye Mo, Yubo Zhang, Xiaoyong Dai, Shu Liu

**Affiliations:** 1 Department of Breast Surgery, The Affiliated Hospital of Guizhou Medical University, Guiyang, China; 2 Department of Breast Surgery, GuiYang Maternal and Child Health Care Hospital, Guiyang, China; 3 Department of Clinical Medicine, Guizhou Medical University, Guiyang, China; 4 Department of Physiology, School of Medicine; Guangdong-Hongkong-Macau CNS Regeneration Institute, Key Laboratory of CNS Regeneration (Ministry of Education); State Key Laboratory of Bioactive Molecules and Druggability Assessment, and Guangdong Province Key Laboratory of Pharmacodynamic Constituents of TCM and New Drugs Research, Jinan University, Guangzhou, China; 5 Colledge of Medical Technology, Guiyang Healthcare Vocational University, Guiyang, China; 6 Synorg Biotechnology (Shenzhen) Co. Ltd., Shenzhen, China; 7 Institute of Biopharmaceutical and Health Engineering, Tsinghua University Shenzhen International Graduate School, Shenzhen, China; 8 Guangxi University of Chinese Medicine, Nanning, China

**Keywords:** autophagy, carbonic anhydrase IX, garciniagifolone A, PI3K/Akt/mTOR pathway, pyroptosis, TNBC

## Abstract

The *Garcinia multiflora*. is not only a delicious fruit that can be consumed directly, but also contains abundant medicinal components in anti-inflammatory and anti-tumor treatments. The objective of this study was to clarify the anti-tumor properties and the underlying mechanism of garciniagifolone A (GA), a polycyclic polyprenylated acylphloroglucinol derived from the edible fruits of Garcinia multiflora, in triple-negative breast cancer (TNBC). Herein, we demonstrated that GA exhibited inhibitory effects on TNBC organoids growth, and suppressed the proliferation, colony formation, migration, and invasion of TNBC cells *in vitro*. Moreover, GA demonstrated a significant suppression of TNBC growth *in vivo* without apparent toxicity, and it augmented the anti-tumor efficacy of PTX in both TNBC organoids and xenograft models. GA specifically bound with carbonic anhydrase IX (CA9), a target overexpressed in TNBC and linked to poor prognosis, as confirmed by molecular docking, surface plasmon resonance, and cellular thermal shift assays. Mechanistically, GA binding to CA9 suppresses the PI3K/AKT/mTOR pathway, inducing early-stage autophagy initiation evidenced by increased LC3B-II/I ratio and autophagosomes. However, GA concurrently blocked autophagic flux by inhibiting autophagosome-lysosome fusion, leading to p62 accumulation and reactive oxygen species (ROS) overproduction. Elevated ROS by GA activated the JNK pathway and triggered NLRP3 inflammasome assembly, resulting in caspase-1-mediated cleavage of gasdermin D (GSDMD) and subsequent pyroptosis, alongside caspase-3-dependent apoptosis. Collectively, GA exerted its potent anti-TNBC activity by targeting CA9 to dysregulate autophagy and induce ROS-mediated pyroptosis/apoptosis, presenting a promising low-toxicity therapeutic strategy.

## 1 Introduction

The triple-negative breast cancer (TNBC), defined by the absence of estrogen receptor (ER), progesterone receptor (PR), and human epidermal growth factor receptor 2 (HER2), accounts for 15% of breast cancer cases and carries the poorest prognosis among subtypes (Derakhshan and Reis-Filho, 2022). TNBC exhibits high genomic heterogeneity and aggressive clinical behavior. At the advanced stage, the cure rate for stage III TNBC is mostly below 70%, while stage IV is often incurable (Maughan et al., 2010; Woolston, 2015). The first-line treatment for TNBC remains cytotoxic chemotherapy using agents such as anthracyclines and taxanes (Ribeiro et al., 2022). The side effects of chemotherapy, including alopecia, nausea, cardiotoxicity, and neurotoxicity, can severely impact patients’ quality of life (Minotti et al., 2004). Therefore, identifying highly effective and low-toxic anticancer agents for TNBC treatment is crucial.

*Garcinia multiflora* Champ. ex Benth. (Clusiaceae) is a versatile species esteemed both as a food source and a medicinal herb. The fruit of G. multiflora, characterized by its yellowish-green pigmentation and a subtly acidic taste, when utilized in its fresh fruits. In traditional medicine, the therapeutic efficacy of the fruit, seeds, and bark of G. multiflora has been acknowledged for the management of ailments, such as bleeding, inflammation and pain (Cao et al., 2024; Kpahe Ziéhi Fidele and Ackah, 2022; Tan et al., 2020). Modern pharmacological studies have elucidated the plant’s potential to exert anti-inflammatory, anti-neoplastic, and antiviral effects (Chen et al., 2009; He et al., 2019; Lima et al., 2024). In our previous study, we discovered that several novel polyprenylated benzophenones, isolated from the fruits of G. oblongifolia (another species belonging to the Garcinia genus), exhibited significant cytotoxic activity against nasopharyngeal carcinoma cells through activating autophagy (Wu et al., 2022). However, the anti-tumour effects and mechanism of Garcinia multiflora in TNBC are still unclear.

Autophagy is a cellular “self-eating” process that engulfs cytosolic proteins and organelles into autophagosomes and delivers them to lysosomes for degradation, thereby maintaining cellular homeostasis (Levy et al., 2017). Autophagy is pivotal in the processes of tumor initiation, growth, and metastasis, and regulation of autophagy is regarded as a significant approach in cancer treatment (Debnath et al., 2023). Notably, the PI3K/AKT/mTOR signaling pathway is a central negative regulator of autophagy, and its inhibition is a well-established strategy to relieve mTORC1-mediated suppression and initiate autophagosome formation (Jiang et al., 2025). However, the functional outcome of autophagy induction is determined by the completion of the entire process, known as autophagic flux. The blockade of autophagic flux, preventing the degradation of cargo, leads to the accumulation of autophagy substrates like p62 and damaged organelles, which can become a source of cellular stress and promote cell death. Critically, the accumulation of damaged mitochondria due to impaired autophagic flux is a major contributor to the excessive production of reactive oxygen species (ROS) (Dong et al., 2023). ROS are considered key elements in NLRP3 activation. This elevated ROS level can then induce the assembly of the NLRP3 inflammasome, and then recruits and cleaves Caspase-1, which in turn cleaves gasdermin D (GSDMD) into its active N-terminal fragment (GSDMD-NT). GSDMD-NT oligomerizes to form pores in the plasma membrane, leading to pyroptosis, a lytic and inflammatory form of programmed cell death, characterized by cell swelling, membrane rupture, and release of proinflammatory cytokines like IL-1β (Wang et al., 2024).

Tumor organoids as an emerging biological model offers distinct advantages over two-dimensional (2D) cell line cultures and animal model (Rauth et al., 2021; Xia et al., 2019). Cancer cell lines lack the ability to maintain the heterogeneity of the original tumor and changes in the tumor microenvironment. Patient-derived tumor xenografts (PDX) are closer to natural tumors but are labor intensive, costly, and have variable efficiency in high-throughput screening (Lee et al., 2018; Sachs et al., 2018). Tumor patient biopsies and surgical specimens can be developed into tumor organoids that closely mimic the original tumor tissues, and can serve as a valuable model for cancer biology study and drug discovery (Piraino et al., 2024). Bhatia S et al. constructed TNBC-derived organoids, wich exhibited signatures of aggressive MYC-driven and basal-like breast cancers (Bhatia et al., 2022).

In this study, we isolated a polycyclic polyprenylated acylphloroglucinol derivative (PPAP), garciniagifolone A (GA), from the fruit of Garcinia multiflora. GA could inhibit TNBC cells growth without obvious toxicity both *in vitro* and *in vivo*. Mechanistically, we found that GA bound specifically with carbonic anhydrase IX (CA9) to induce autophagy by blocking the PI3K/AKT/mTOR pathway. Moreover, GA increased p62 levels to block autophagic flux, and promoted ROS overproduction to activate NLRP3/GSDMD-related pyroptosis and apoptosis in TNBC cells.

## 2 Materials and methods

### 2.1 Extraction and isolation of GA

The dried whole part of G. multiflora fruits (25.0 kg) was extracted with 95% ethanol at room temperature, and obtained a crude residue (2.3 kg) under reduced pressure. This residue was added to chromatography with silica gel column, and eluted with CH_2_Cl_2_-CH_3_OH (100:0 to 0:100, v/v) to yield six fractions (Fr. A-F). Fr. A was further applied to a silica gel CC with PE/EtOAc (1000:0 to 0:1000, v/v) to afford five subfractions (Fr. A1-A5). Fr.A3 (49.4 g) was alternately separated several times using ODS column chromatography (CH_3_OH-H_2_O), small-bore column chromatography(CH_3_OH-H_2_O), and Sephadex LH-20 gel column chromatography to achieve crude separation, and finally the compound GA (43.0 mg) was obtained by preparative HPLC (MeOH/H_2_O, 70:30, v/v), and the purity was 99%.

### 2.2 Cell lines

Human cell lines MCF-7, BT549, MDA-MB-231, SKBR3, BT474, CAL51, MCF10A and HUVECs were purchased from iCell company (Shanghai, China). BT474 cells were maintained in 10% FBS RPMI-1640 medium (Gibco, C11875500BT). MCF10A cells were cultured in a specific epithelial medium (Procell Co., Ltd, CL-0525). The others were cultured in 10% FBS DMEM (Gibco, C11995500BT) medium. All medium were further supplemented with a penicillin-streptomycin solution (double antibody), 100× (Procell Co., Ltd, PB180120). Cells were cultured in an incubator at 37 °C and 5% CO_2_.

### 2.3 Cell Counting Kit-8 (CCK8) assay

GA was dissolved in DMSO to prepare a 50 mM stock solution, which was stored at −80 °C and diluted with culture medium to the desired working concentrations. The final concentration of DMSO was kept constant at 0.1% (v/v) in all treatment groups, including the vehicle control groups. Each experimental condition was performed in triplicate wells, and the entire experiment was independently repeated three times. The MDA-MB-231 and BT549 (8 × 10^3^ cells/well) were incubated in 96-well plates overnight. The cells were treated with GA (8 μM) alone or with the 740 Y-P 25 μM (MCE, HY-P0175) and LY294002 20 μM (MCE, HY-10108) for 24 h. Cell viability was measured using the Cell Counting Kit-8 (Beyotime, C0038). Specifically, 10 µL of CCK-8 solution was added to each well, and the plates were incubated for 2 h. After incubation, the absorbance was measured at 450 nm using a microplate reader. Data analysis and calculations were performed using GraphPad Prism nine software.

### 2.4 Cell apoptosis analysis

The final concentration 0.1% (v/v) of DMSO was the vehicle control groups. The entire experiment was independently repeated three times. The MDA-MB-231 and BT549 cells (3 × 10^5^ cells/well) were seeded in 6-well plates and incubated for 24 h. Following the treatment of GA (4 μM, 8 μM) for another 24 h, the cells were trypsinized with 0.25% Trypsin (1×) (EDTA-free), resuspended in 1× binding buffer, and stained using the TransDetect® AV/PI Cell Apoptosis Detection Kit (Transgen, FA101-02). The process of apoptosis was examined utilizing FlowJo software (Beckman FACSCalibur Flow Cytometer, Brea, CA, USA).

### 2.5 EdU staining assay

The MDA-MB-231 and BT549 cells were seeded into 96-well plates at a density of 6 × 10^3^ cells per well, and incubated for 24 h. The final concentration 0.1% (v/v) of DMSO was the vehicle control groups. After 24 h treatment with control reagent or GA (4 μM, 8 μM), the sample was assessed using the special kit (Ribobio, C10310-1), incorporating five-ethyl-2′-deoxyuridine (EdU) 50 μM. The entire experiment was independently repeated three times. Finally, photographs of the stained cells were captured using fluorescence microscopy (Nikon, Japan).

### 2.6 Colony formation assay

The MDA-MB-231 and BT549 cells (5 × 10^2^ cells/well) were seeded in 6-well culture plates overnight. Following treatment for 13 days, these samples were immobilized using 4% PFA (Beyotime, P0099-500 mL) for 20 min, then stained with 0.2% crystal violet solution (Beyotime, C0121-100 mL) for 30 min. Afterward, the colonies were gently washed, photographed, and counted.

### 2.7 Transwell assay

The MDA-MB-231 and BT549 cells were seeded at a density of 8 × 10^4^ cells per well in the upper chamber of transwell inserts (Biosharp, BS-15-GJM). Different concentrations of GA (to avoid toxic effects, set at 3 μM and 6 μM) were added to serum-free medium (200 μL), while the lower chamber was filled with medium containing 20% FBS as a chemical attractant (700 μL). For the invasion assay, a high-concentration Matrigel basement membrane matrix (Corning, 354,234) was pre-cooled and diluted with serum-free medium at a volume ratio of 1:8. 100 μL of the diluted solution was evenly applied to the surface of the Transwell polycarbonate membrane and solidified in a 37 °C incubator for 30 min. The subsequent steps were the same as those for the cell migration experiment. After treating the cells with GA for 24 h, the medium in the upper chamber was removed, and cells that did not penetrate the matrix and residual Matrigel were gently removed using a pre-cooled PBS-moistened cotton swab. Then, the chapper were fixed and stained with 4% PFA and 0.5% crystal violet for 30 min, respectively. The entire experiment was independently repeated three times. After image acquisition, the ImageJ software was used to quantify the number of migrated or invaded cells.

### 2.8 Tube formation assay

Matrigel was stored at 4 °C in a refrigerator for 24 h. It was then placed in 48-well plates and incubated at 37 °C for 1 h to solidify. Next, 1.5 × 10^4^ HUVECs cells were seeded in each well containing different concentrations of GA and incubated for 4 h. The entire procedure was performed while avoiding the formation of air bubbles.

### 2.9 TNBC organoids construction

In this study, we used tissue samples from five diagnosed and untreated TNBC patients (Supplementary Table S1) in Guiyang Maternal and Child Health Hospital. Biopsy samples were obtained with approval from Guiyang Maternal and Child Health Hospital Ethics Committee (No. 2023–99). Specimens were transported to the laboratory in a organizational protective solution (Miltenyi Biotec, 130–100–008) at 4 °C. They were cut into small pieces after washing three times in the pre-cooled PBS. A portion of this tissue was fixed in 4% PFA (Biosharp, BL539A) for pathological analysis. The remaining tissue was used to extract primary cells to construct organoids. The chopped tissues were incubated with Cebrary®Tissue Digestion Solution (Yeasen, 41423ES10) on a shaker for 30 min at 37 °C. Cells were centrifuged at 300 *g* about 5 min and then gently resuspended in pre-cooled Matrigel (Corning, 356,234) at 4 °C. The cell density of organoids was 1.5 × 10^4^ cells/μl. The hydrophobic phase of the e-fluoride solution and the matrigel liquid stream were sheared into mono-dispersed micro-droplets with a diameter of about 650 μm, using the organoid 3D fabrication platform (Zhang W et al., 2024). The cells were transferred to a 6 cm dish containing 2.5 mL MasterAim® Breast Cancer Organoid Complete Medium (Aimingmed, 10–100–002). For passaging, the organoids were digested with TrypLE Express enzyme (Gibco, 12605028) for 5 min at 37 °C. After digestion, the cells were centrifuged for 5 min at 300 g, and then resuspended in Matrigel and obtained the organoids microsphere through the organoid 3D fabrication platform. To evaluate the role of GA in TNBC organoids, organoids were collected after culturing for 5 days, resuspended in pre-chilled Matrigel, and embedded in 96-well plates at a density of 1 × 10^4^ cells/10 µL Matrigel. After 24 h, they were treated with various concentrations of GA (3.1 µM, 6.3 µM, 12.5 µM, 25 μM, 50 μM, 100 µM) for another 6 days. The viability and imaging of the TNBC organoids were then assessed using the CellTiter-Glo Luminescent Cell Viability Assay Kit (Promega, G7572) and AM/PI Live/Dead Double Staining Kit (Beyotime, C1371M) according to the manufacturers' instructions.

### 2.10 RNA sequencing

When the cell density reached 70%, cells were treated with 0 or 8 μM GA for 24 h. At the time of collection, cells were washed with PBS and lysed by adding 1 mL of Trizol (Beyotime, R0016), followed by pipetting to ensure thorough mixing. The processed samples were stored at −80 °C overnight and then transported on dry ice to test (Chengqi, Guangzhou).

### 2.11 The mcherry-GFP-LC3B adenovirus transfection

The AdPlus-mCherry-GFP-LC3B kit (Beyotime, C3012-1 mL) was used to measure autophagic flux after cell infection. In brief, The MDA-MB-231 and BT549 cells (1 × 10^4^ cells/dish) were inoculated on confocal dishes (Biosharp, BS-15-GJM) for 24 h. Then, mCherry-GFP-LC3B adenovirus at a multiplicity of infection of 20 was diluted into a single culture solution and added to the cells according to the instructions. When the cellular density attained approximately 80%, the cells underwent treatment with various concentrations of GA (4 μM, 8 μM) alone or with the Torin 1 250 nM (MCE, HY-13003) and CQ 50 μM (MCE, HY-17589A) for a duration of 24 h. Subsequently, the cells were immobilized with 4% PFA for 30 min at ambient temperature, followed by exposure to an anti-fluorescence quenching mounting droplet. After the incubation period, the fluorescence of red and yellow puncta was visualized and documented using laser confocal microscopy. The entire experiment was independently repeated three times.

### 2.12 Western blotting

Cells were collected and lysed on ice with RIPA (Beyotime, P0013B) to extract proteins, and the BCA Kit (Bioss, C05-02001) was used to detect protein concentration. The electrophoretic separation proteins were transferred to PVDF (MerkMillipore, IPVH00010), and the membranes were incubated with 5% BSA in TBST buffer (Leagene, PW0026) for 1 h to block non-specific binding. The membranes were then cut based on the molecular weight (MW) of the target protein and incubated with the primary antibody overnight at 4 °C with gentle shaking. On the following day, the membranes were washed with TBST buffer and then incubated with an HRP-conjugated secondary antibody for 1 h. After washing three times for 30 min, protein bands were detected using Omni-ECL™ reagent (Epizyme, SQ201) and imaged with a GelView9000 Lite chemiluminescence imaging system (BLT, Guangzhou, China). The antibodies were used in the Western blotting analysis were in Supplementary Table S2. The entire experiment was independently repeated three times.

### 2.13 The ROS assay

The MDA-MB-231 and BT549 cells (3 × 10^5^ cells/well) were seeded for 24 h, and then exposed to various concentrations of GA (4 μM, 8 μM) for another 24 h. To assess ROS levels, a detection kit from Yeasen (50101ES01) was utilized. The cells were stained with 2′,7′-dichlorofluorescein diacetate (DCFH-DA) for 30 min at 37 °C. Subsequently, the levels of ROS were measured employing both a fluorescence microscope and a flow cytometer. Cells were first gated on the FSC-A/SSC-A scatter plot to exclude debris and define the live cell population. Within the single cell gate, the fluorescence intensity was shown as histogram in the FITC/GFP channel (corresponding to DCFH-DA oxidation) was analyzed for both treated and control groups. The final results are presented as the population of cells with a high fluorescence intensity of ROS and the ROS generation rate. The fluorescent probe used was DCFH-DA, which was excited by a 488 nm laser, and its emission was detected at 530 ± 30 nm. The entire experiment was independently repeated three times.

### 2.14 Immunofluorescence (IF) analysis

The processed cell or tissue samples were placed in culture plates, fixed by 4% PFA at room temperature, removed the fixative and washed three times with PBS, and treated with 0.2% Triton X-100 for 20 min at room temperature. Cells were blocking with 5% BSA at room temperature. Next, the primary antibody was added, and incubated in a wet box at 4 °C overnight. Primary antibodies included: Ki67 (Huabio, HA721115), p63 (Huabio, ET1610-44), MUC1 (Huabio, HA601142), CK8/18 (ThermoFisher, MA5-14088), CK7 (Proteintech, 17513-1-AP), CA125 (Huabio, ET1611-74), EpCAM (Proteintech, 66316-1-Ig), HER2(Huabio, HA721178), PR (Huabio, ET1702-24) and ER (Proteintech, 20698-1-AP). Then, fluorescent secondary antibody (Huabio, HA1121, HA1126, 1:500 dilution) was added, and incubated for 1 h at room temperature. Cell nuclei were then labeled by incubation with DAPI stain for 5 min and photographed using a laser confocal microscope.

### 2.15 Molecular docking

Molecular docking analysis was performed to investigate the binding mode between GA and CA9 using AutoDock Vina (version 1.0.2). The crystal structure of human CA9 in complex with a reference inhibitor (ligand code: ALA377) was retrieved from the RCSB Protein Data Bank (PDB ID: 5FL4, resolution 1.82 Å). The three-dimensional structure of GA was obtained from the PubChem database. Protein preparation was carried out using AutoDockTools. All crystallographic water molecules were removed except those directly involved in coordination with the catalytic Zn^2+^ ion. Hydrogen atoms were added, and Gasteiger charges were assigned. The Zn^2+^ ion located in the active site was explicitly retained and treated as a rigid metal center, preserving its native tetrahedral coordination geometry. Protonation states of ionizable residues were assigned assuming physiological pH (7.4), and histidine residues were protonated based on their local hydrogen-bonding environments. The GA structure was converted into a three-dimensional conformation and energy-minimized prior to docking. File format conversions were performed using Open Babel software. Ligand preparation, including assignment of rotatable bonds and Gasteiger charges, was conducted using AutoDockTools, with protonation states set to reflect physiological conditions. The most favorable docking pose was visualized using Discovery Studio 2021 Client and PyMOL (version 2.2.0).

### 2.16 Surface plasmon resonance (SPR)

The SPR experiments were performed using a Biacore T200 instrument (GE Healthcare) at 25 °C, with HBS-EP running buffer (10 mM HEPES, pH 7.4; 150 mM NaCl; 3 mM EDTA; 0.5% (v/v) surfactant P20; and 5% DMSO). The CA9 protein (Cusabio, CSB-EP614990HUc0) was coupled to the surface of CM5 chip biosensor (Cytiva, 29104988) in 10 mM sodium acetate (pH 4.5). To determine the optimal coupling conditions, the pH values yielding the highest response were selected, and the corresponding sodium acetate buffer was used for the coupling process. Similarly, the optimal protein concentration was determined based on the initial response values. Following the coupling template instructions, EDC/NHS solution, ethanolamine hydrochloride solution, sodium hydroxide solution, and CA9 protein solution were sequentially loaded into the sample rack, and the coupling parameters were set accordingly. GA compound solutions at varying concentrations were injected over the CA9-immobilized sensor surface at a flow rate of 20 μL/min for 120 s to allow binding. Dissociation was monitored by injecting running buffer for an additional 120 s. The sensorgram data were processed using Biacore T200 Evaluation Software version 1.0 (GE Healthcare) to calculate the dissociation constant (KD) and analyze the binding kinetics. Affinity and kinetic models were fitted to the sensorgram data obtained from multiple GA concentrations to determine the KD and gain insights into the binding interactions between GA and CA9.

### 2.17 The cellular thermal shift assay (CETSA)

MDA-MB-231 or BT549 cells were collected and lysed on ice using RIPA lysis buffer (Beyotime, P0013B) supplemented with a protease and phosphatase inhibitor cocktail (Beyotime, P1050). The cell lysates were centrifuged at 15,000 × g for 15 min at 4 °C to remove insoluble debris. The supernatant (whole cell lysate) was collected, and the protein concentration was determined. The lysates were then divided into two aliquots and incubated with either GA or an equal volume of DMSO for 30 min at room temperature. Following incubation, the lysates were aliquoted into PCR tubes and heated at the indicated temperatures for 3 min. The heated samples were centrifuged at 20,000 × g for 20 min at 4 °C to separate the soluble protein fraction from the aggregates. The supernatant was analyzed by Western blotting to assess the thermal stability of the CA9 protein.

### 2.18 qRT-PCR assay

Total RNA was extracted using Trizol (Beyotime, R0016). After quantification, RNA was reverse transcribed using Hifair® II 1st Strand cDNA Synthesis SuperMix for qPCR (Yeasen, 11123ES60) according to the manufacturer’s instructions. The mRNA levels of CA9 and GAPDH (used as an internal control) were detected using qPCR Master Mix (Promega, A6002) through qRT-PCR instrument (Analytikjena, qTOWER3G). The specified genes were amplified using the primer sequences in Supplementary Table S3. The qPCR reaction was performed using a two-step amplification protocol: initial pre-denaturation at 95 °C for 2 min, followed by 40 cycles of amplification with each cycle consisting of denaturation at 95 °C for 15 s and annealing/extension at 60 °C for 60 s. A melting curve analysis was then conducted by gradually increasing the temperature from 60 °C to 95 °C. Gene expression was calculated using the 2^−ΔΔCt^ method. The entire experiment was independently repeated three times.

### 2.19 CA9-siRNA transfection

The MDA-MB-231 and BT549 cells (2 × 10^5^ cells/well) were seeded into 6-well plates. After 24 h, when the cell density reached approximately 50%, the cells were transfected using the CA9-siRNA kit (MCE, HY-RS01776) according to the manufacturer’s, instructions. The specified genes were amplified using the primer sequences in Supplementary Table S3. The final concentration of siRNA is 40 nmol/L. The transfection complexes were incubated for 48 h were harvested.

### 2.20 Animal model

The experiments were consented by the Ethics Review Committee of Shenzhen Graduate School of Peking University (ER-0042–030). Female BALB/c nude mice, aged 6 weeks and sourced from the Guangdong Medical Laboratory Animal Center, and allocated into five groups. The MDA-MB-231 cells (3 × 10^6^ cells/100 μL PBS) were injected subcutaneously in the left axilla. Following tumor formation, animals were randomized using a computer-generated sequence to ensure balanced baseline conditions. When the tumor reached approximately 3 mm^3^ in size, PTX, a common chemotherapeutic agent for TNBC, was used as a positive control. The animals were randomly divided into five groups: saline (control), GA (10 mg/kg), PTX (5 mg/kg), GA (20 mg/kg), and a combination of GA (20 mg/kg) and PTX (5 mg/kg). Mice were administered the drug via intraperitoneal injection every 2 days. Body weight and tumor size were recorded throughout the study. Tumor volume was calculated using the formula: length × width^2^ × 0.5. After a 23-day treatment period, the mice were euthanized, and tumors, blood, and various organs were collected for further analysis. During the treatment and tumor measurement phases, a single-blind protocol was strictly implemented. The operator was unaware of group assignments, and data analysis was performed in a blinded manner. Furthermore, the experiment adhered to predefined animal health monitoring indicators, clear inclusion/exclusion criteria, and a humane endpoint.

### 2.21 HE and IHC staining

The HE and IHC staining were conducted as previously described^29^. Standard immunohistochemistry protocols were then followed, utilizing monoclonal antibodies to stain the tumor sections. The SAB assay and 3′3′-diaminobenzidine tetrahydrochloride (DAB) were performed using predetermined optimal concentrations of primary antibodies. Additionally, paraffin-embedded tumor tissue and vital tissue (including the lung, kidney, heart, liver, and spleen) were stained with HE for histological studies.

### 2.22 TUNEL staining

Apoptosis *in vivo* was determined using TUNEL staining (i.e., riboAPO One-Step TUNEL Apoptosis Kit (Red) (Ribobio, C11026-2)). The samples were added a mixture of TdT and dUTP and incubated at 37 °C for 1 h away from light. Following this, the cell nuclei were incubated DAPI for a duration of 10 min. The samples were observed and photographed with a fluorescence microscope.

### 2.23 Toxicology assay

At the time of mouse execution, orbital blood samples were collected and placed in 1.5 mL centrifuge tubes. These samples were then centrifuged at 12,000 g for 15 min at 4 °C, and the supernatants were collected. The detection kits sourced from Najjcbio, encompassed CRE, BUN, LDH, AST, ALT and AKP.

### 2.24 Statistical analysis

The data points are presented as mean ± (SD). To compare the groups, we employed one-way ANOVA followed by Tukey’s multiple comparisons test (Graphpad Software, USA). Differences were deemed statistically significant at *p < 0.05, **p < 0.01, ***p < 0.001, and ****p < 0.0001 levels.

## 3 Results

### 3.1 GA inhibits the proliferation of TNBC cells and organoids *in Vitro*

The GA was obtained from the fruits of Garcinia multiflora, and the chemical formula as shown in Supplementary Figures S1a. The Chromatogram, HR-ESI-MS, IR spectrum and UV spectrum of GA were showed in Supplementary Figure S1b-e. The proton signals were analyzed by ^1^H NMR spectrum were summarized as follows: [δ_H_ 1.71 (3H, s), 1.69 (3H, s), 1.67 (6H, s), 1.66 (3H, s), 1.60 (3H, s), 1.59 (3H, s), 1.56 (3H, s), 1.18 (3H, s), 1.12 (3H, s)] corresponding to nine methyl protons; [δ_H_ 5.03 (1H, t, J = 6.3 Hz), 4.96 (1H, d, J = 7.7 Hz), 4.84 (1H, m), 4.71 (1H, s), 4.60 (1H, s)] corresponding to five olefinic protons; and [δ_H_ 7.20 (1H, s), 6.66 (1H, d, J = 7.8 Hz), 7.26 (1H, d, J = 8.0 Hz)] corresponding to three aromatic protons (Supplementary Figure S1f). Further analysis of the ^13^C NMR signals suggests a molecular formula of C_33_H_48_O_6_, with Ω = 10 (Supplementary Figure S1g). The DEPT-135 spectrum showed that the 38 carbon signals of GA consist of nine primary carbons, five secondary carbons, 10 tertiary carbons, and 14 quaternary carbons. Analysis of the chemical shift characteristics identified four carbonyl groups, one benzene ring, and three pairs of double bonds (Supplementary Figure S1h). The HNMR and HPLC data were also provided in Supplementary Figures S1i, j. The NMR data was presented in Supplementary Table S4.

**FIGURE 1:**
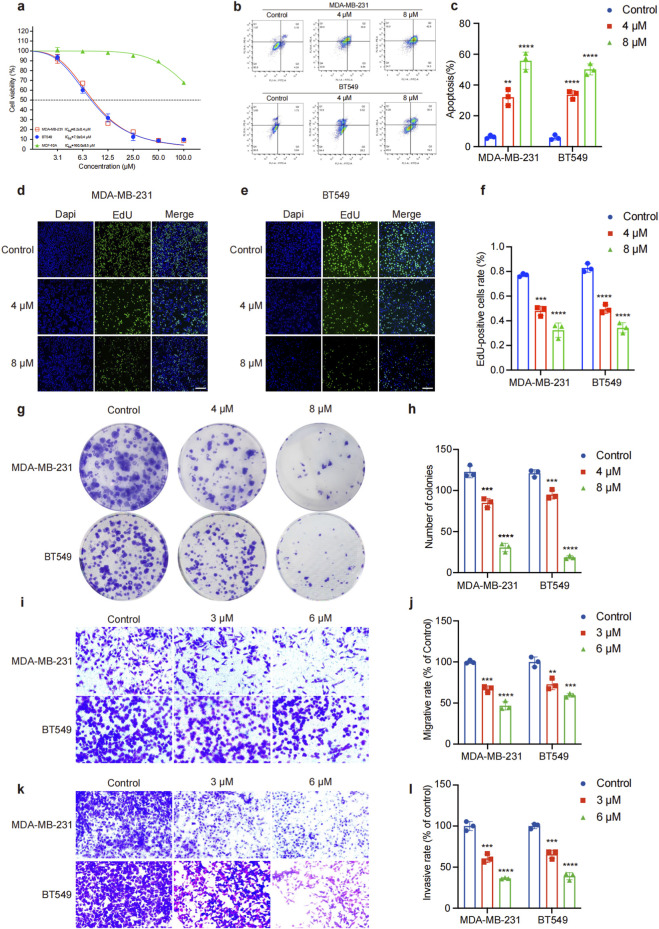
GA inhibits the proliferation, colony formation, migration and invasion of TNBC cells *in vitro*. **(a)** The identification of anti-TNBC effects of GA by using MDA-MB-231, BT549 and MCF-10A cells. **(b,c)** Flow cytometry was used to detect cell apoptosis in MDA-MB-231 and BT549 cells with different concentrations of GA for 24 h **(d–f)** EdU staining of MDA-MB-231 and BT549 cells by GA treatment for 24 h. Scale bar: 100 μm. **(g,h)** The colony formation of MDA-MB-231 and BT549 cells by GA treatment. **(i,j)** Cell migration ability of MDA-MB-231 and BT549 cells was detected using transwell assay. Scale bar: 100 μm. **(k, l)** Cell invasion ability of MDA-MB-231 and BT549 cells was detected using transwell assay. Scale bar: 100 μm. Data are presented as mean ± SD. n = 3. ^*^p < 0.05, ^**^p < 0.01, ^***^p < 0.001, and ^****^p < 0.0001 when compared with control group.

To verify the anti-TNBC effects of GA, we used the TNBC cell lines for verification. The cytotoxic effects of GA on TNBC cells were evaluated using a CCK8 assay. As shown in Figure 1a, the proliferation of the TNBC cells MDA-MB-231 and BT549 were obviously inhibited by GA, with the IC_50_ value as 8.2 ± 0.4 μM and 7.9 ± 0.4 μM, respectively. Moreover, the IC_50_ value of GA in MCF-10A was 160 ± 8.5 μM, which indicated that GA had low toxicity in normal mammary epithelial cells. The flow cytometry results demonstrated that GA treatment significantly induced both early and late apoptosis in TNBC cells when compared with control group (Figures 1b,c). In addition, EdU staining revealed that GA notably inhibited DNA synthesis in TNBC cells (Figures 1d–f). Colony formation assays further confirmed that GA markedly suppressed the colony formation of MDA-MB-231 and BT549 cells, indicating its ability to impede long-term cell survival and clonogenicity (Figures 1g,h). The HUVEC angiogenesis assay showed that GA exerted a dose-dependent inhibition on angiogenic activities in endothelial cells (Supplementary Figures S2-d). The transwell assays revealed that GA suppressed the migratory and invasive capabilities of MDA-MB-231 and BT549 cells in a dose-dependent manner, further supporting its potential as an anti-metastatic agent (Figures 1i–l).

We then validated the anti-TNBC effects of GA by using TNBC organoids. As shown in Supplementary Figure S3a, TNBC organoids were observed with tumor spheres increasing in size. HE staining demonstrated that the cells’ proliferation in organoids increased with culture duration, and the cell morphology in organoids presented large nuclear volume and an oval-shaped irregular shape as parental tumor tissue. The immunofluorescence (IF) staining showed a low expression of CK7 and p63 while high expression of MUC1, CK8/18, EpCAM and CA125 both in TNBC organoids and parental tumor tissues (Supplementary Figure S3b) when compared with paracancerous tissues and related organoids (Supplementary Figure S4). Furthermore, the absence of ER, PR, HER2 was also confirmed in TNBC organoids (Supplementary Figure S3c). In contrast, CA125 is lowly expressed, while ER and PR are highly expressed in paracancerous tissues and related organoids (Supplementary Figure S4). These results demonstrated that TNBC organoids preserved the histological structure and biomarkers of parental tumors. To further assess the anti-TNBC efficacy of GA, five organoids derived from different TNBC patients were constructed. The IC_50_ values of GA in TNBC organoids ranged from 7.3 to 8.1 µM, indicating that GA possessed significant anti-tumor activity against TNBC (Supplementary Figure S3d). Together, these findings suggested that GA effectively inhibited TNBC cells growth, migration, metastasis and angiogenesis *in vitro*.

### 3.2 GA induces autophagy by blocking PI3K/AKT/mTOR signaling pathway in TNBC cells

**FIGURE 2:**
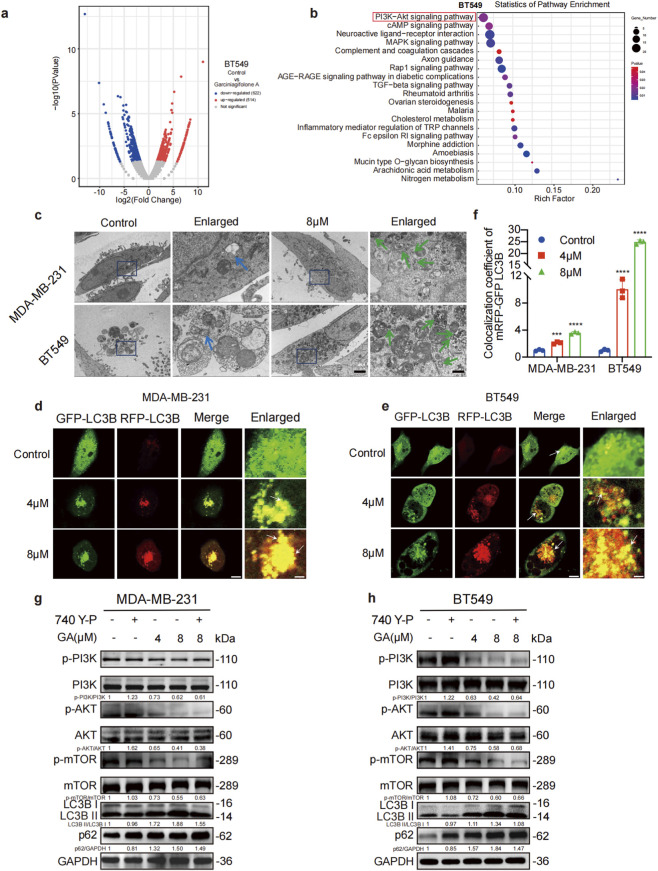
GA induces autophagy via blocking PI3K/AKT/mTOR signaling pathway in TNBC cells. **(a)** The volcano plot of RNA-seq displayed the differentially expressed genes (DEGs) between the control group and the GA treated group. **(b)** KEGG data analysis revealed that the PI3K/AKT/mTOR signaling pathway was significantly inhibited after GA treatment. **(c)** Transmission electron microscope (TEM) images of GA-treated cells. Arrows indicated the autophagosomes. Scale bar: 2.5 μm and 0.5 μm in normal and enlarged, respectively. **(d,e)** Confocal image of MDA-MB-231 and BT549 cells transfected with mRFP-GFP-LC3B plasmid. Scale bar: 25 μm and 5 μm in normal and enlarged, respectively. Arrow: the colocalization of GFP-LC3B and RFP-LC3B. **(f)** Colocalization area statistics in MDA-MB-231 and BT549 cells. **(g,h)** Levels of the p-PI3K, PI3K, p-AKT, AKT, p-mTOR, mTOR, LC3B and p62 after treatment with GA in the presence or absence of 740 Y-P (25 μM) for 24 h were measured by Western blotting. Data are presented as mean ± SD. n = 3. ^*^p < 0.05, ^**^p < 0.01, ^***^p < 0.001, and ^****^p < 0.0001 when compared with control group.

Many recent studies have demonstrated that autophagy was closely related with TNBC cells growth (Jiang et al., 2023; Zhang et al., 2023). We first performed RNA sequencing and KEGG analyses to find the genes and pathway differences between the control and GA-treated groups. The most significant change of genes between control and GA-treated group were enriched in PI3K/AKT/mTOR signaling pathway both in BT549 and MDA-MB-231 (Figures 2a,b; Supplementary Figure S5). The PI3K/AKT/mTOR pathway was validated to regulate cell autophagy. To investigate whether GA could inhibit TNBC cells autophagy, the transmission electron microscopy (TEM) was used. As shown in Figure 2c, the autophagosomes in GA-treated group exhibited characteristic double-membrane structures (blue and green arrow) containing cytoplasmic components, indicating that autophagy was effectively activated by GA. The GFP-RFP-LC3 plasmids was transfected into TNBC cells to monitor the dynamic changes of autophagic flux. The GFP-RFP-LC3 plasmid is a dual-fluorescence reporter system designed to dynamically monitor autophagic flux in living cells. In this construct, LC3 (a key autophagy marker) is fused to both GFP (green fluorescent protein) and acid-stable RFP/mCherry (red fluorescent protein) in tandem. During autophagosome formation, the neutral pH environment preserves both GFP and RFP fluorescence, resulting in yellow puncta (GFP and RFP overlap). However, upon fusion of autophagosomes with lysosomes to form autolysosomes, the acidic lysosomal environment quenches GFP fluorescence due to its pH sensitivity, while RFP remains stable. Consequently, an increase in the proportion of yellow puncta indicates the obstruction of autophagic flow, and an mature autolysosomes exhibit only red puncta. As shown in Figures 2d–f, the green fluorescence was stable, and yellow puncta was significantly increased both in MDA-MB-231 and BT549 cells after GA treatment. As shown in Supplementary Figure S6, the Torin1 could enhace the inhibition effects of GA in the autophagy flux, while CQ and NAC all reversed the effecs both in MDA-MB-231 and BT549 cells. Meanwhile, we found that the activator (740 Y-P) of PI3K/AKT/mTOR pathway could reverse the anti-tumor effects of GA, while inhibitor (LY294002) enhanced the inhibitory effects of GA both in MAD-MB-231 and BT549 cells (Supplementary Figure S7). These results suggested that the PI3K/AKT/mTOR pathway plays a key role in Garciniagifolone A-induced cell death. GA promoted the early stage of autophagy, while blocking the autophagic flux through inhibiting fusion of autophagosomes with lysosomes.

To further investigate whether GA could regulate autophagy through blocking PI3K/AKT/mTOR signaling pathway, the Western blot analysis was performed. As shown in Figures 2g,h, the ratios of p-PI3K/PI3K, p-AKT/AKT, and p-mTOR/mTOR were significantly reduced, while the LC3B-II/LC3B-I ratio and p62 level were all increased in GA-treated cells compared to control. In contrast, activation of PI3K/AKT/mTOR pathway by using PI3K activator 740Y-P could specially reversed the effects of GA both in PI3K/AKT/mTOR pathway and autophagy related markers in combine group (Figures 2g,h). These data suggested that GA could activate early stage of autophagy through suppressing PI3K/AKT/mTOR pathway, and inhibited fusion of autophagosomes with lysosomes to block autophagic flux.

### 3.3 GA induces ROS overproduction by activating the JNK pathway

**FIGURE 3:**
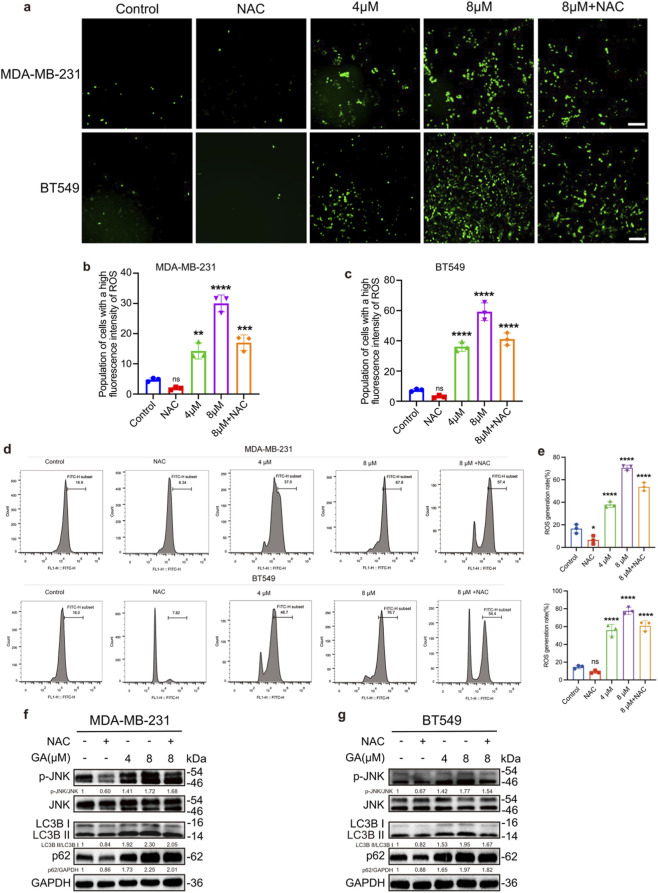
GA induces ROS overproduction via activating JNK pathway. **(a)** DCFH-DA fluorescent image of ROS in MDA-MB-231 and BT549 cells. Scale bar: 200 μm. **(b,c)** Quantitative analysis of ROS production. **(d,e)** ROS production measured by flow cytometry. **(f,g)** Levels of the p-JNK, JNK, LC3B and p62 after treatment with GA in the presence or absence of NAC (5 mM) for 24 h measured by Western blotting. Data are presented as mean ± SD. n = 3. ^*^p < 0.05, ^**^p < 0.01, ^***^p < 0.001, and ^****^p < 0.0001 when compared with control group.

Blocking autophagy flux and increasing p62 can promote ROS production (Liu et al., 2023). To assess whether GA could induce an increase in ROS, the 2′,7′-dichlorodihydrofluorescein diacetate (DCFH-DA) probe was used. The fluorescence data revealed a significant elevation in ROS levels in both MDA-MB-231 and BT549 cells following GA treatment (Figures 3a–c). Flow cytometry analysis further confirmed that GA significantly augmented ROS production in TNBC cells (Figures 3d,e). Additionally, Western blot analysis showed that the phosphorylation level of JNK was significantly increased by GA in a dose-dependent manner (Figures 3f,g). Pre-treatment with NAC, an ROS scavenger, could reduce the level of ROS and JNK phosphorylation, and reversed the promotion effects of GA in ROS production (Figures 3f,g). The NAC significantly decreased the levels of LC3-II/LC3-I and p62, which indicated that NAC restored autophagy function through decreasing the ROS. Moreover, the NAC also reversed the effects of GA in autophagy flux, confirming that GA promoted the ROS overproduction through blocking the autophagic flux (Figures 3f,g). These findings suggested that GA induced ROS overproduction potentially via the activation of the JNK signaling pathway.

### 3.4 GA promotes TNBC cells pyroptosis and apoptosis by activating the NLRP3/caspase-1/GSDMD/caspase-3 pathway

**FIGURE 4:**
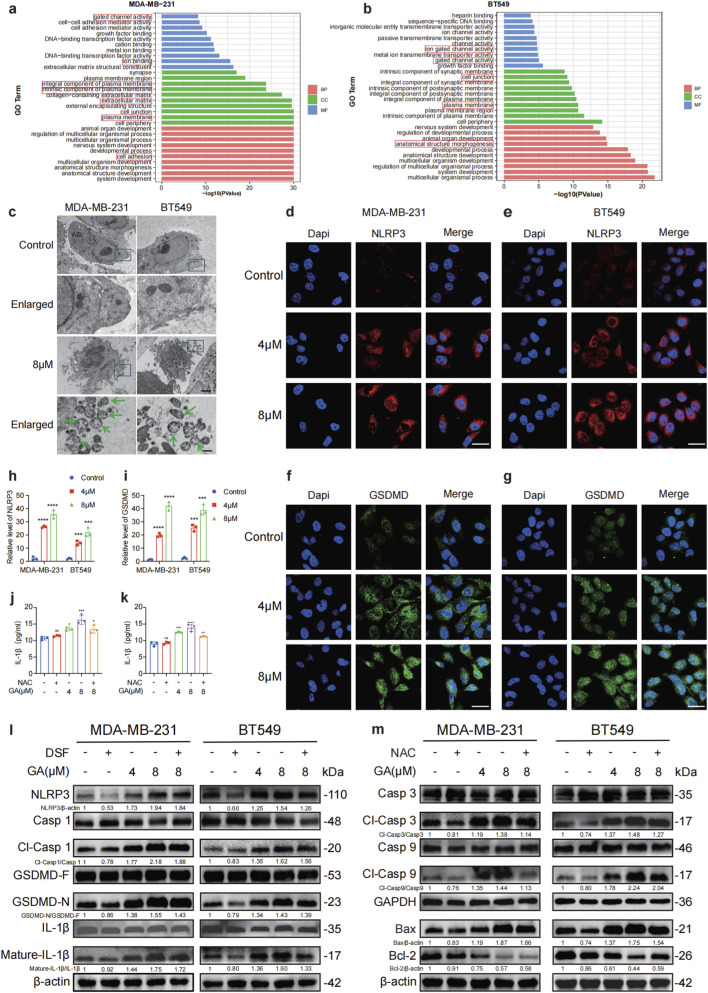
GA promotes TNBC cells pyroptosis and apoptosis via activating NLRP3/caspase-1/GSDMD/caspase-3 pathway. **(a,b)** Gene Ontology (GO) enrichment analysis was performed on the DEGs to identify significantly enriched biological processes (BP), molecular functions (MF), and cellular components (CC) in MDA-MB-231 and BT549 cells. **(c)** Transmission electron microscope (TEM) images of GA-treated MDA-MB-231 and BT549 cells. Arrows indicate pyroptotic cells. Scale bar: 2.5 μm and 0.5 μm in normal and enlarged, respectively. **(d–g)** The protein levels of NLRP3 and GSDME was detected by immunofluorescence. Scale bars: 50 μm. **(h,i)** Colocalization area statistics in MDA-MB-231 and BT549 cells. **(j,k)** The levels of IL-1β were analyzed by ELISA. **(l)** The levels of pyroptosis-related proteins (NLRP3, caspase-1, cleaved caspase-1, GSDMD-F, GSDMD-N,IL-1β and Mature- IL-1β) were measured by Western blotting after treating with GA for 24 h in the presence or absence of DSF (5 μM). **(m)** The levels of apoptosis-related proteins (caspase-3, cleaved caspase-3, caspase-9, cleaved caspase-9, Bax, and Bcl-2) were measured by Western blotting after treating with GA for 24 h in the presence or absence of NAC (5 mM). Data are presented as mean ± SD. n = 3. ^*^p < 0.05, ^**^p < 0.01, ^***^p < 0.001, and ^****^p < 0.0001 when compared with control group.

Recent studies have underscored the pivotal role of ROS in triggering the activation of the NLRP3 inflammasome and subsequently driving the release of key pro-inflammatory cytokines, such as IL-3, IL-1β, and TNF, across a range of inflammatory disorders (Luo et al., 2022; Wu et al., 2018). We performed a gene ontology (GO) enrichment analysis and found significant associations with cellular components related to the cell membrane in terms of biological processes, molecular functions, and cellular components. These findings indicated that the implicated genes may be involved in membrane-associated cellular pathways (red box) (Figures 4a,b). The TEM data showed swell and large plasma membrane blebs morphology (green arrow) in the GA-treated cells (Figure 4c). Subsequently, the immunofluorescence staining showed an increase in NLRP3 and GSDMD, further verifying the occurrence of cell pyroptosis (Figures 4d–i). Furthermore, the accumulation of IL-1β was observed in a dose-dependent manner in the supernatant of MDA-MB-231 and BT549 cell culture medium, as measured by ELISA, and the secretion of IL-1β was reduced after the addition of NAC, further confirming that ROS induced the generation of NLRP3 inflammasome (Figures 4j,k). The Western blot analysis revealed a substantial increase in the expression of N-GSDMD fragments (GSDMD-N), cleaved caspase-1, NLRP3, and cleaved IL-1β proteins following GA treatment. Notably, this effect was reversed by the pyroptosis inhibitor DSF (Figure 4l). In addition, the bax, cleaved caspase-3, and cleaved caspase-9 were all increased, while Bcl-2 downregulated after GA treatment (Figure 4m). Using the ROS scavenger NAC could reverse the effects of GA in apoptosis related proteins, which indicated that GA promoted TNBC cells apoptosis via ROS overproduction. Collectively, these findings suggested that GA induced TNBC cells pyroptosis and apoptosis by activating the NLRP3/caspase-1/GSDMD/caspase-3 signaling pathway.

### 3.5 GA inhibits TNBC growth by targeting CA9

**FIGURE 5:**
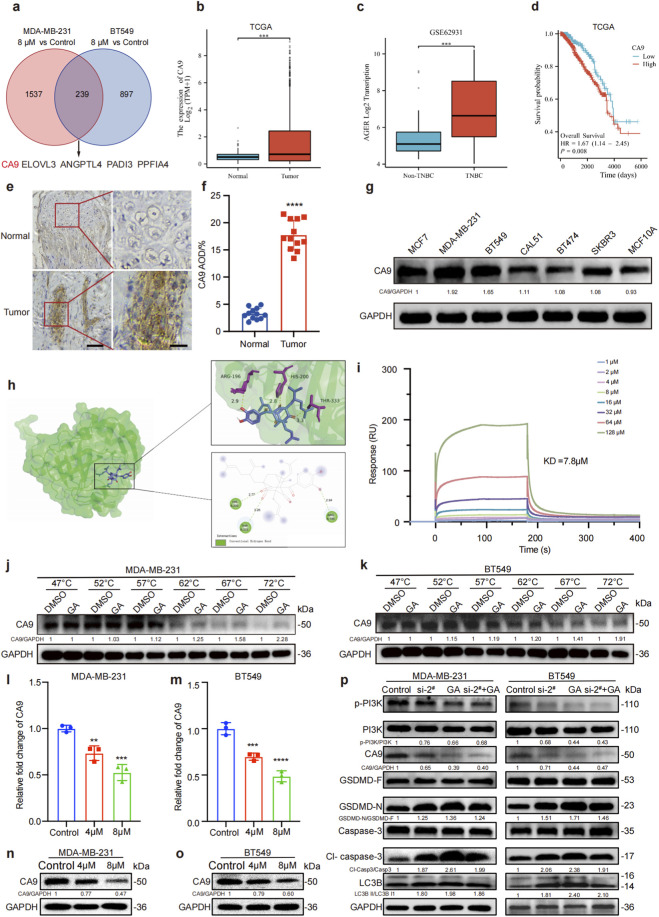
GA inhibits TNBC growth via targeting CA9. **(a)** Venn diagram showing the number of differentially expressed genes in the comparison between MDA-MB-231 and BT549. **(b,c)** TCGA and GEO databases show that CA9 is highly expressed in TNBC. **(d)** Correlation analysis between TNBC patient prognosis and CA9 expression. **(e)** IHC staining of TNBC patient-derived cancer tissue and adjacent tissue. Scale bar: 100 μm. **(f)** The quantitative analysis of CA9. **(g)** The protein levels of CA9 in cell lines were measured by Western blotting. **(h)** The predicted binding mode of GA with CA9. **(i)** GA binds to CA9 protein as shown by SPR. **(j,k)** CETSA analysis of CA9 combined with GA. **(l,m)** CA9 mRNA levels after GA 24 h treatment by qRT-PCR. **(n,o)** CA9 protein levels after GA 24 h treatment by Western blotting. **(p)** The related protein levels of siRNA-CA9 TNBC cells after GA 24 h treatment by Western blotting. Data are presented as mean ± SD. n = 3. ^*^p < 0.05, ^**^p < 0.01, ^***^p < 0.001, and ^****^p < 0.0001 when compared with control group.

The lack of effective targets is the main reason for TNBC poor prognosis, and finding novel and effective targets has became a critical need. To explore the potential anti-TNBC target of GA, we utilized Venn diagram analysis to identify differential gene expression and found that 239 genes were significantly differentially expressed between the GA-treated and control group. Notably, CA9 exhibited the most pronounced changes, which suggested its potential as a key target in the therapeutic action of GA against TNBC (Figure 5a). To investigate whether CA9 could serve as a potential therapeutic target for TNBC, we utilized data from the Cancer Genome Atlas (TCGA) database. Analysis revealed that CA9 expression was markedly elevated in tumor cells compared to normal tissue cells (Figure 5b). Similarly, the Gene Expression Omnibus (GEO) database showed that CA9 was highly expressed in TNBC compared with non-TNBC (Figure 5c). Prognostic analysis demonstrated a significant association between elevated CA9 expression and poor clinical outcomes in cancer patients (p = 0.008) (Figure 5d). IHC staining demonstrated that CA9 was overexpressed in TNBC compared with adjacent tissues (Figures 5e,f). Western blot showed that CA9 was highly expressed in MDA-MB-231 and BT549 cells (Figure 5g). These results indicated that CA9 was highly expressed in TNBC, and may be closely associated with TNBC poor prognosis.

Docking results revealed that GA could establish a stable structure with CA9 complex, exhibiting a binding energy ΔG of −7.7 kcal/mol for CA9 (PDB code: ALA377) (Figure 5h). The SPR results showed that GA exhibited a notable binding affinity to the CA9 protein, with an estimated dissociation constant (KD = 7.8 μM) (Figure 5i), indicating a specific and measurable interaction. To further substantiate CA9 as a molecular target of GA, the CETSA was conducted, which revealed that GA not only bound to CA9 but also significantly enhanced its thermal stability, suggesting a direct stabilizing effect on the protein structure (Figures 5j,k). To investigate the effects of GA on the expression of CA9, we examined the mRNA and protein levels of CA9 in TNBC cells after GE treatment using qRT-PCR and Western blot. As shown in Figures 5l–o, the mRNA and protein levels of CA9 were all decreased in a dose-dependent manner in GA-treated group. Subsequently, we used siRNA to knockdown the CA9 in MDA-MB-231 and BT549 cells, and the knockdown efficiency was verified by qRT-PCR and Western blot (Supplementary Figures S8a-d). The results showed that si-2^#^ had the highest knockdown efficiency, and Si-2^#^ was selected for constructing the CA9 knockdown model in subsequent experiments. In addition, the cell viability was significantly decreased after CA9 knockdown and GA treatment (Supplementary Figures S8e, f). In order to verify whether GA induced autophagy and pyroptosis were mediated by targeting CA9, the Western blot was performed after CA9 knockdown by si-2^#^. As shown in Figure 5p, the expression level of p-PI3K were significantly downregulated, while GSDMD-N, cleaved caspase-3, and LC3B-II/LC3B-I ratio were all markedly upregulated by GA. In contrast, knockdown of CA9 could specially moderate the effects of GA on above protein levels. These findings demonstrated CA9 as a potential therapeutic target for GA in the treatment of TNBC.

### 3.6 GA inhibits TNBC cells growth without observable toxicity *in Vivo*

**FIGURE 6:**
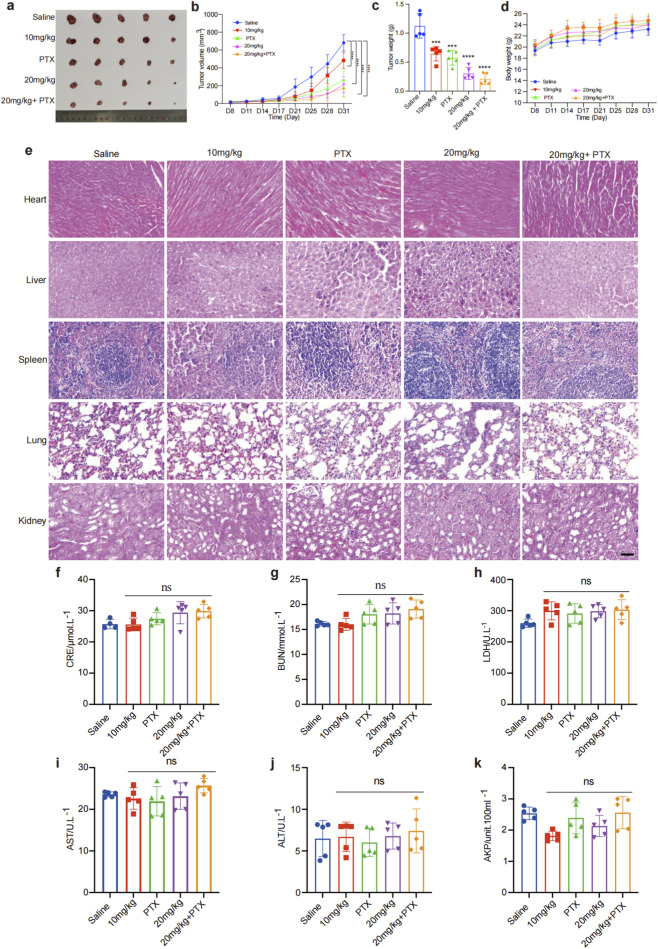
GA inhibits TNBC cells growth *in vivo*. **(a)** The photography of tumor tissues. **(b)** The tumor volume growth curve. **(c)** The tumor weight statistics. **(d)** Body weight statistics. **(e)** HE staining of the main organs (heart, liver, spleen, lung, and kidney). Scale bar: 100 μm. **(f–k)** Serum level of CRE, BUN, LDH, AST, ALT and AKP, n = 5. Data are presented as mean ± SD. n = 5. ^*^p < 0.05, ^**^p < 0.01, ^***^p < 0.001, and ^****^p < 0.0001 when compared with control group.

To further investigate the anti-TNBC potential of GA *in vivo*, the MDA-MB-231 cells xenograft model were established. The animal were randomly divided into five groups: saline (control), GA (10 mg/kg), PTX (5 mg/kg), GA (20 mg/kg), and a combination of GA (20 mg/kg) and PTX (5 mg/kg). The tumor volume and tumor weight were all decreased by GA in a dose-dependent manner (Figures 6a–c). The inhibitory effect of combine group was better than PTX alone group, which indicated that GA could enhance the anti-tumor effects of PTX *in vivo*. Analysis of body weight changes revealed no significant differences among the five groups, indicating that GA did not cause general toxicity (Figure 6d). Additionally, histopathological examination of major organs (heart, liver, spleen, lungs, and kidneys) through HE staining showed no evidence of tissue damage in any of the treatment groups compared to the control (Figure 6e). Biochemical assays of liver and kidney function indicators, including CRE, BUN, LDH, AST, ALT and AKP, revealed no significant alterations, further suggesting that GA induced no significant systemic toxicity (Figures 6f–k). Collectively, these findings demonstrated that GA exerted a potent anti-TNBC effect *in vivo* without observable toxicity.

### 3.7 GA induced autophagy and pyroptosis of TNBC cells *in Vivo*

**FIGURE 7:**
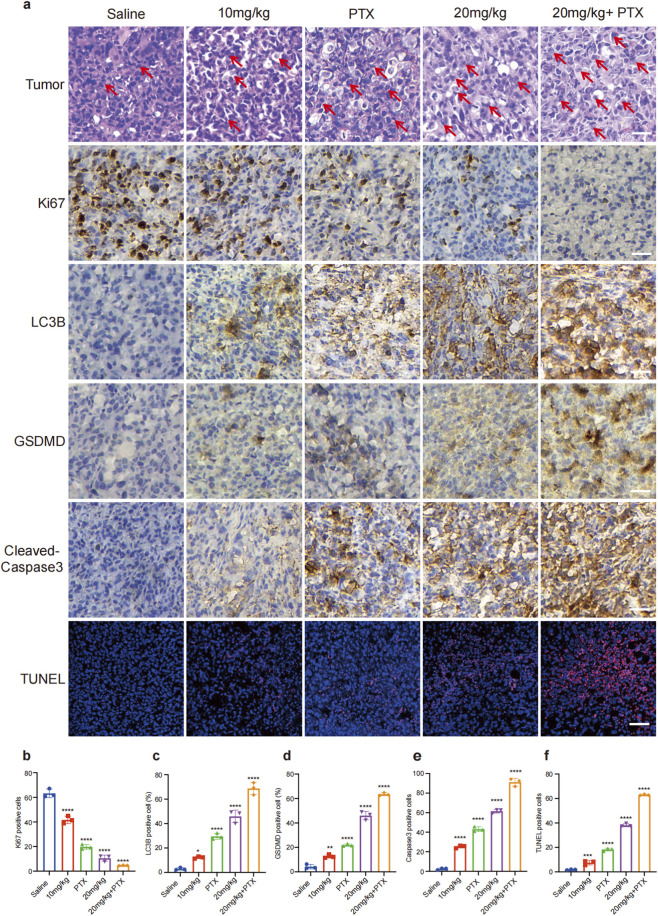
GA promotes autophagy and pyroptosis of TNBC cells *in vivo*. **(a)** HE, IHC and TUNEL staining. Scale bar: 50 μm. **(b–f)** The quantitative analysis of IHC and TUNEL. Data are presented as mean ± SD. n = 3. ^*^p < 0.05, ^**^p < 0.01, ^***^p < 0.001, and ^****^p < 0.0001 when compared with control group.

To verify whether GA could promote TNBC cells autophagy and apoptosis *in vivo*, the tumor tissues were analyzed using HE and IHC staining. As depicted in Figures 7a, b HE staining demonstrated that tumors in the control group exhibited diffuse growth, marked by a densely packed, disorganized, and loosely arranged cellular architecture. In contrast, the tumors in the GA treatment group exhibited a lighter staining of both the cytoplasm and the nuclei, a reduction in nuclear division phenomena, and a decreased nuclear-cytoplasmic ratio (red arrow). The IHC analysis revealed that GA treatment markedly reduced Ki67 expression, while elevating the levels of LC3B, GSDMD and cleaved caspase-3 (Figures 7a,c–e). Additionally, TUNEL staining indicated a pronounced increase in apoptotic cell numbers in the GA-treated group compared to the control group (Figures 7a–f). Collectively, these findings confirmed that GA induced autophagy and pyroptosis in TNBC cells *in vivo*.

### 3.8 GA enhances the anti-tumor effects of PTX in TNBC organoids

**FIGURE 8:**
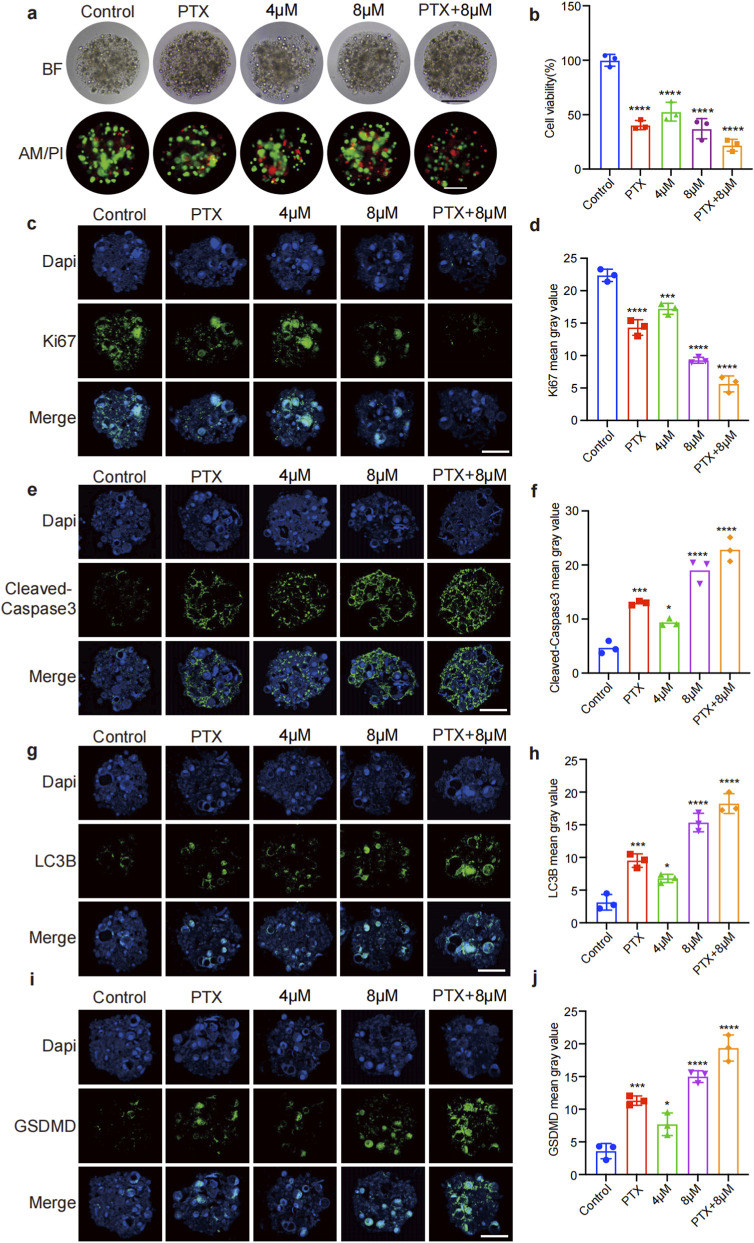
GA enhances the anti-tumor effects of PTX in TNBC organoids. **(a)** The AM/PI staining of TNBC organoids. Scale bar: 100 μm. **(b)** The cell viability of TNBC organoids by CTG assay. **(c–f)** Immunofluorescence detection of changes in Ki67, cleaved caspase-3, LC3B and GSDMD. Scale bar: 100 μm. **(g–j)** Immunofluorescence quantification statistics. Data are presented as mean ± SD. n = 3. ^*^p < 0.05, ^**^p < 0.01, ^***^p < 0.001, and ^****^p < 0.0001 when compared with control group.

Chemotherapy resistance remains the primary cause of mortality in TNBC (Ferrari et al., 2022). To evaluate whether GA could enhance the sensitivity of TNBC cells to chemotherapeutic agents, TNBC organoids were employed. The AM/PI staining revealed a dose-dependent increase in dead cells (indicated by red fluorescence) upon GA treatment (Figure 8a). The cell Titer-Glo 3D assays further demonstrated that GA potentiated the anti-tumor effects of PTX in TNBC organoids (Figure 8b). Immunofluorescence staining showed a significant reduction in Ki67 expression, coupled with a marked upregulation of cleaved-caspase-3 in the combination treatment group when compared PTX alone group (Figures 8c–f). Additionally, the levels of LC3B and GSDMD were all obviously elevated in the combine groups (Figures 8g–j). These findings suggested that GA could enhance the anti-tumor efficacy of PTX in TNBC organoids, likely through the activation of autophagy, pyroptosis, and apoptosis pathways.

## 4 Discussion

The most novel finding of this study is to elucidate the anti-TNBC mechanism and therapy target of GA. Our study confirmed GA as a promising therapeutic agent for TNBC with minimal side toxicity by using several preclinical models, including TNBC cell lines, xenograft mice model and organoids. We note that the relatively limited variation in IC_50_ values observed across the five patient-derived organoid (PDO) models, while suggesting consistent potency of GA, may also reflect certain limitations of this study. This consistency could be attributed to two factors: first, the shared high expression of GA’s target, CA9, across diverse TNBC subtypes—a hypoxia-inducible pathway that may underlie a common vulnerability beyond genomic heterogeneity; and second, the possibility that the PDOs used, all derived from untreated primary TNBC patients, may represent a subset with a shared basal-like phenotype and thus exhibit more uniform responses. Nevertheless, this homogeneity also highlights a limitation in the scope of our models, as organoids derived from treatment-resistant tumors or rarer TNBC subtypes (e.g., luminal androgen receptor subtype) were not included. Therefore, future studies with expanded sample sizes and more diverse TNBC models are necessary to fully evaluate the breadth of GA’s efficacy and its applicability across the spectrum of TNBC heterogeneity.

The proposed mechanism, wherein GA binding to CA9 leads to suppression of the PI3K/AKT/mTOR pathway, is supported by emerging evidence of functional crosstalk between hypoxia-associated proteins and core oncogenic signaling networks. CA9 is a hypoxia-inducible transmembrane enzyme crucial for maintaining intracellular pH homeostasis in the acidic tumor microenvironment, thereby promoting tumor cell survival, migration, and invasion (Swietach et al., 2009). Importantly, CA9 is not merely a passive biomarker of hypoxia but an active signaling node. Recent studies indicate that CA9 can engage in functional interactions with key oncogenic pathways. Specifically, hypoxia-induced CA9 expression has been shown to promote EGFR stabilization and activation, which is a major upstream regulator of the PI3K/AKT axis (Lock et al., 2013). More directly, a study in renal carcinoma cells demonstrated that CA9 knockdown resulted in reduced phosphorylation of AKT, suggesting a tangible regulatory link between CA9 expression and AKT signaling activity (Salaroglio et al., 2018). While the precise molecular mechanism connecting CA9 to PI3K/AKT/mTOR in TNBC is still being elucidated, our data showed that GA binding to CA9 leads to its downregulation and a consequent decrease in p-AKT and p-mTOR levels, which provide strong experimental evidence for this functional connection in our models. This represents a novel and therapeutically relevant mechanism of action for a natural compound in TNBC.

**FIGURE 9:**
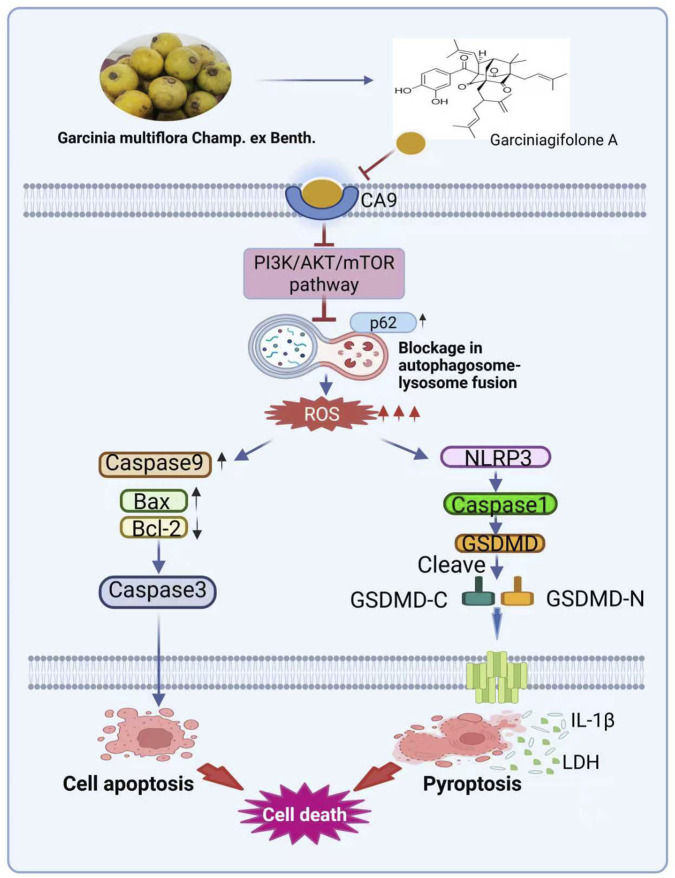
Model of the anti-tumor mechanism of GA in TNBC cells. GA could bind CA9 to induce early stage autophagy by blocking PI3K/AKT/mTOR signaling pathway. Moreover, GA suppressed the autophagic flux and increased the p62 to promote ROS production, which activated NLRP3/caspase-1/GSDMD/caspase-3 related pyroptosis and apoptosis to induce TNBC cells death.

GA could activate early stage of autophagy through inhibiting PI3K/AKT/mTOR pathway, while increasing p62 levels to block autophagic flux, which induced ROS overproduction. The elevated ROS by GA could promote TNBC cells pyroptosis and apoptosis via activating the NLRP3/caspase-1/GSDMD/caspase-3 signaling pathway (Figure 9). We also confirmed the CA9 as the anti-TNBC target of GA by using molecular docking, SPR, and CETSA analysis, and knockdown CA9 could reverse the anti-TNBC effects of GA. CA9 is a tumor-associated cell surface glycoprotein related to hypoxia, and has been identified as a promising new anticancer target in many cancers (Cui et al., 2023; Debreova et al., 2019; Svastova et al., 2012). CA9 can regulate PH homeostasis in the tumor microenvironment, which is important for solid malignancies (Gibadulinova et al., 2020). When hypoxia occurs, apoptosis (Temiz et al., 2021), autophagy (Gul et al., 2018), and pyroptosis (Zhao et al., 2024) are induced. It has been established that CA9 is strongly correlated with adverse clinical outcomes and poor survival rates in TNBC (Ong et al., 2022; Shamis et al., 2023). To explore the relationship between CA9 and TNBC, we conducted a bioinformatics analysis using data from the TCGA and GEO databases. Our analysis revealed that CA9 significantly overexpressed in TNBC and that CA9 overexpression was strongly associated with a poor prognosis in TNBC. Molecular docking and surface plasmon resonance revealed a significant binding affinity between GA and CA9. Additionally, CETSA analysis showed that GA not only bound to CA9 but also stabilized its structure. Experimental validation confirmed that GA not only bound with CA9, but also decreased the mRNA and protein level of CA9 to exert its anti-TNBC therapeutic effects. Moreover, CA9 knockdown significantly reversed the anti-tumor effects of GA both in functional experiments and signal mechanisms. The above results successfully confirmed CA9 as the therapeutic target of GA in TNBC.

Autophagy is a vital cellular process that sustains homeostasis by degrading damaged or unnecessary proteins and organelles. The accumulation of LC3B-II correlates with the formation of autophagosomes and reflects overall autophagic activity. Similarly, p62 serves as another key marker, acting as a selective receptor that aids in the recognition and degradation of substrates. When autophagic flux is impaired, p62 accumulates, as it cannot be degraded in the absence of functional autophagy. Thus, alterations in LC3B and p62 levels offer crucial insights into the efficiency and dynamics of autophagic processes. In our study, Western blotting results demonstrated that GA significantly upregulated the LC3B-II/LC3B-I ratio, thereby promoting autophagy. The GFP/RFP/mCherry autophagic flux assays indicated that while GA facilitated the early stages of autophagy, it impaired the later stage by blocking the fusion of autophagosomes with lysosomes and preventing p62 degradation, leading to its accumulation. Additionally, GA treatment reduced the phosphorylation of mTOR, PI3K, and AKT, suggesting that GA induced autophagy through inhibition of the PI3K/AKT/mTOR pathway. In conclusion, GA initiated early stages of autophagy but inhibited fusion of autophagosomes with lysosomes to block autophagic flux, resulting in elevated p62 level.

The PI3K/AKT/mTOR pathway is a frequently dysregulated oncogenic driver in TNBC, making it a compelling therapeutic target (Ellis and Ma, 2019). Numerous PI3K pathway inhibitors have been developed and evaluated in clinical trials for breast cancer, including pan-PI3K inhibitors (e.g., Buparlisib), isoform-selective inhibitors (e.g., Alpelisib, for PIK3CA-mutant cancers), and dual PI3K/mTOR inhibitors (e.g., Dactolisib). However, their clinical application, particularly in TNBC, has been severely hampered by significant challenges. First, on-target toxicities such as hyperglycemia, rash, and hepatotoxicity are common due to the critical role of PI3K signaling in normal metabolic homeostasis (Zhang H et al., 2024). Second, intrinsic and acquired resistance mechanisms frequently emerge, including feedback activation of alternative pathways (e.g., MAPK/ERK or JAK/STAT), mutations in upstream nodes (e.g., RTKs), or loss of PTEN (Yang et al., 2023). Furthermore, the efficacy of selective PI3Kα inhibitors is limited to a subset of patients with PIK3CA mutations, which are less common in TNBC compared to other subtypes. In our study, we found Garciniagifolone A (GA) as a novel and distinct entity with potential to overcome some of these limitations. Unlike synthetic ATP-competitive inhibitors that directly target the kinase domain, GA is a natural product that binds to CA9, a transmembrane enzyme highly overexpressed in the hypoxic tumor microenvironment of TNBC but with restricted expression in normal tissues. Moreover, GA’s anti-tumor effect is not merely cytostatic but potently cytotoxic, inducing a multi-modal cell death program involving apoptosis, impaired autophagy, and most notably, pyroptosis. This inflammatory cell death may not only effectively kill tumor cells but also potentially stimulate an anti-tumor immune response, a feature lacking in conventional PI3K inhibitors (Wang et al., 2020). While recent efforts continue to develop novel PI3K inhibitors with improved isoform selectivity or combination strategies to mitigate toxicity and resistance (Hao et al., 2025), GA offers a unique approach by targeting a non-kinase, microenvironmental antigen upstream of the pathway, thereby providing a new therapeutic avenue for TNBC treatment.

ROS is a toxic byproduct of cellular metabolism, and is closely correlated with cell autophagy. When autophagy is inhibited, it leads to excessive ROS, which mediates mitochondrial damage (Filomeni et al., 2015; Xing et al., 2022). Our study indicated that upon treatment with GA, the intracellular ROS level was significantly increased, which was reversed by ROS inhibitor, NAC. ROS can promote pore formation downstream of GSDMD cleavage, and is considered as a key element of NLRP3 and caspase-1 activation (Zhou et al., 2018). The activation of caspase-1 enhances the conversion of pro-IL-1β to mature IL-1β and cleaves GSDMD into GSDMD-CT and GSDMD-NT, the latter of which oligomerizes to form cell membrane pores, thereby activating cell pyroptosis. In this study, TEM showed that there was pyroptosis-related cell morphology in the GA-treated cells. Similarly, we found that NLRP3, GSDMD-N, cleaved caspase-1, mature IL-1β, cleaved caspase-9, cleaved caspase-3 and Bax levels were all significantly upregulated after treatment with GA. These results confirmed that GA induced ROS overproduction to activate cell pyroptosis and apoptosis by activating the caspase-1/NLRP3/GSDMD/caspase-3 signaling pathway.

It is well-documented that sustained and complete inhibition of autophagic flux can, in certain contexts, lead to the accumulation of toxic aggregates and damaged organelles, ultimately promoting cell death (Guo et al., 2024; Pan et al., 2019). However, the paradoxical scenario also exists where tumor cells might adapt to partial autophagy inhibition by upregulating alternative survival pathways, such as enhanced antioxidant defense systems (e.g., Nrf2 signaling) to counteract ROS, or mutations in key death receptors and inflammasome components (e.g., NLRP3, caspase-1) to evade pyroptosis. We posit that the unique multi-modal mechanism of GA may inherently mitigate the risk of rapid resistance development. Firstly, GA does not merely inhibit autophagy initiation, which triggers the process while simultaneously blocking its completion. This creates an irreconcilable cellular stress scenario characterized by p62 accumulation and unabated ROS generation, which is inherently difficult for cells to adapt. Secondly, the induction of inflammatory pyroptosis via GSDMD cleavage presents a direct and potent lytic cell death pathway that may bypass classical apoptosis resistance mechanisms commonly found in TNBC. The release of pro-inflammatory cytokines (e.g., IL-1β) could further stimulate an anti-tumor immune response, potentially providing a long-term protective effect against relapse. Nevertheless, future studies are essential to rigorously investigate potential resistance mechanisms. These will include generating GA-resistant cell lines to identify compensatory pathways, evaluating the efficacy of GA in combination with antioxidants (like NAC) or inhibitors of pyroptosis to confirm the dependence on these mechanisms, and exploring synergistic strategies with immunotherapies that could leverage the immunogenic nature of GA-induced cell death.

## 5 Conclusion

In summary, our study assessed the therapeutic potential of GA in TNBC using organoids, cell lines, and xenograft models. GA significantly inhibited TNBC cells growth without obvious side toxicity, both *in vitro* and *in vivo*, and could enhance the anti-tumor effect of PTX. Mechanistically, GA bound CA9 to induce autophagy by blocking the PI3K/AKT/mTOR pathway. However, GA suppressed the autophagic flux and increased p62 to promote ROS production, which activated caspase-1/NLRP3/GSDMD/caspase-3-related pyroptosis and apoptosis to induce TNBC cell death. Our study provides a new theoretical basis for the anti-breast cancer effect of GA. In our short-term preclinical model, it was observed that GA did not induce significant systemic toxicity (such as weight loss or acute organ damage) while exerting potent therapeutic effects, indicating a favorable initial therapeutic window. Although these findings support the rationality of further investigating GA as a potential therapeutic candidate, we recognize that definitive conclusions regarding its clinical applicability and safety still need to be verified through comprehensive long-term toxicological studies and future clinical trials.

## Data Availability

The original contributions presented in the study are included in the article/Supplementary Material, further inquiries can be directed to the corresponding authors.
