# Supplementary material for: Garciniagifolone A, derived from Garcinia multiflora fruits, inhibits triple-negative breast cancer cells and organoids growth by targeting CA9
Source: Front Cell Dev Biol. 2026 Mar 11;14:1767397. doi: 10.3389/fcell.2026.1767397 (PMC13013538; doi:10.3389/fcell.2026.1767397)
Supplement: Supplementary file 1 [file Supplementaryfile1.docx]

**Supplemental materials**

**Table. S1 Information of five TNBC patients**

| **Number** | **Age** | **Tumor size(cm)** | **Tumor location** | **IHC** |
| --- | --- | --- | --- | --- |
| Patient 1 | 43 | 2.5×1.7×2.4 | right upper outer quadrant of the breast | ER(-), PR(-), HER2(-), Ki67 (40%) |
| Patient 2 | 50 | 2.9×1.8×2.3 | right lower outer of the breast | ER(-), PR(-), HER2(-), Ki67 (30%) |
| Patient 3 | 49 | 3.2×2.5×2.6 | right central breast region | ER(-), PR(-), HER2(-), Ki67 (30%) |
| Patient 4 | 47 | 2.8×2.3×1.7 | upper left breast | ER(-), PR(-), HER2(-), Ki67 (50%) |
| Patient 5 | 55 | 3.5×2.3×2.7 | medial part of the left breast | ER(-), PR(-), HER2(1+), Ki67 (60%) |

**Table. S2 Antibody list in Western blotting**

| **Antibody** | **Manufacturer** | **Product Code** | **Concentration** |
| --- | --- | --- | --- |
| p-PI3K | Thermo Fisher Scientific | PA5-118549 | 1:1000 |
| PI3K | Thermo Fisher Scientific | PA5-110625 | 1:1000 |
| p-AKT | ABclonal | AP1208 | 1:1000 |
| AKT | ABclonal | A17909 | 1:1000 |
| p-mTOR | ABclonal | AP0115 | 1:1000 |
| p-JNK | ABclonal | AP0631 | 1:1000 |
| JNK | ABclonal | A4867 | 1:1000 |
| NLRP3 | ABclonal | A21906 | 1:1000 |
| GSDMD | ABclonal | A18281 | 1:1000 |
| IL-1β | ABclonal | A16288 | 1:1000 |
| Caspase-3 | ABclonal | A19664 | 1:1000 |
| LC3B | ABclonal | A19665 | 1:1000 |
| p62 | ABclonal | A11483 | 1:1000 |
| Bax | ABclonal | A20227 | 1:1000 |
| Bcl-2 | ABclonal | A19693 | 1:1000 |
| GAPDH | ABclonal | A19056 | 1:25000 |
| Cleaved-caspase-3 | Cell Signaling Technology | 94530 | 1:1000 |
| β-actin | Cell Signaling Technology | 19069 | 1:5000 |
| Caspase-9 | Abcam | ab185719 | 1:1000 |
| CA9 | Abcam | ab184006 | 1:1000 |
| Cleaved-caspase-9 | Affinity Biosciences | AF4000 | 1:1000 |
| Cleaved-caspase-1 | Affinity Biosciences | AF4022 | 1:1000 |
| mTOR | Zen Bio | 380411 | 1:1000 |
| Caspase-1 | ServiceBio | GB11383 | 1:1000 |
| HRP Conjugated AffiniPure Goat Anti-rabbit IgG (H+L) | Boster Biological Technology | BA1055 | 1:5000 |
| HRP Conjugated AffiniPure Goat Anti-mouse IgG (H+L) | Boster Biological Technology | BA1050 | 1:5000 |

**Table. S3 The primer sequence**

| **Gene** | Forward (5′-3′) | Reverse (5′-3′) |
| --- | --- | --- |
| CA9 | ACCTGGTGACTCTCGGCTACAG | CAGCCAGGCAGGAATTCAGC |
| GAPDH | ATTTGGTCGTATTGGGCG | CTCGCTCCTGGAAGATGG |
| si-1^#^ | GCCGCUACUUCCAAUAUGAGG | UCAUAUUGGAAGUAGCGGCUG |
| si-2^#^ | GGCUGCUGGUGACAUCCUAGC | UAGGAUGUCACCAGCAGCCAG |
| si-3^#^ | GACUGUGUUUAACCAGACAGU | UGUCUGGUUAAACACAGUCCA |

**Table. S3 NMR data of Garciniagifolone A (400 MHz)^a^**

| **Position** | **Garciniagifolone A (CDCl_3_)** | |
| --- | --- | --- |
|  | ***δ*_H_/ppm (Hz)** | ***δ*_C_/ppm** |
| 1 | - | 76.9 |
| 2 | - | 202.0 |
| 3 | - | 79.5 |
| 4 | - | 201.4 |
| 5 | - | 68.5 |
| 6 | 2.50 (m), 2.35 (m) | 44.2 |
| 7 | 1.64 (overlap) | 47.7 |
| 8 | - | 53.9 |
| 9 | - | 203.9 |
| 10 | - | 193.0 |
| 11 | - | 127.5 |
| 12 | 7.20 (s) | 116.1 |
| 13 | - | 144.1 |
| 14 | - | 148.7 |
| 15 | 6.66 (d, 7.8) | 114.2 |
| 16 | 6.47 (d, 8.0) | 124.5 |
| 17 | 2.11 (m), 1.78 (dd, 14.6, 4.1) | 33.3 |
| 18 | 2.78 (m) | 42.8 |
| 19 | - | 149.9 |
| 20 | 4.60 (s), 6.71 (s) | 112.9 |
| 21 | 1.59 (s) | 18.2 |
| 22 | 2.05 (m) | 33.5 |
| 23 | 5.03 (t, 6.3) | 122.9 |
| 24 | - | 132.2 |
| 25 | 1.56 (s) | 18.2 |
| 26 | 1.60 (s) | 25.9 |
| 27 | 2.50 (m), 2.35 (m) | 23.2 |
| 28 | 4.84 (t, 7.0) | 119.6 |
| 29 | - | 134.6 |
| 30 | 1.69 (s) | 18.3 |
| 31 | 1.67 (s) | 26.1 |
| 32 | 1.18 (s) | 22.7 |
| 33 | 1.12 (s) | 23.3 |

**
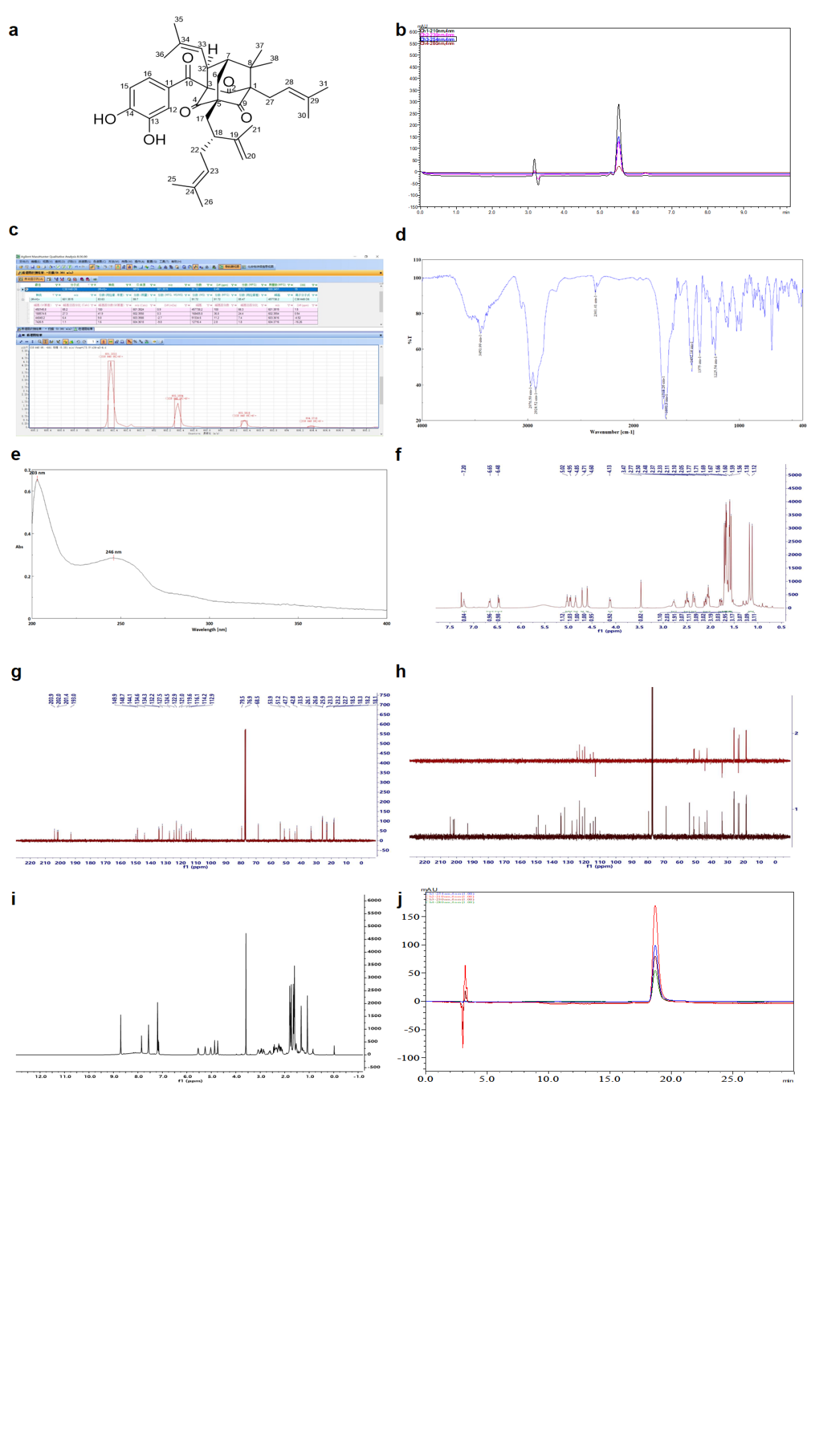
**

**FIGURE S1** The extraction and isolation of GA. (a) Chemical structure of GA. (b) Chromatogram of GA. (c) HR-ESI-MS of GA. (d) IR spectrum of GA. (e) UV spectrum of GA. (f) 1H NMR spectrum of GA. (g) ^13^C NMR spectrum of GA. (h) DEPT-135 spectrum of GA. (i) HNMR of GA. (j) HPLC of GA.

**
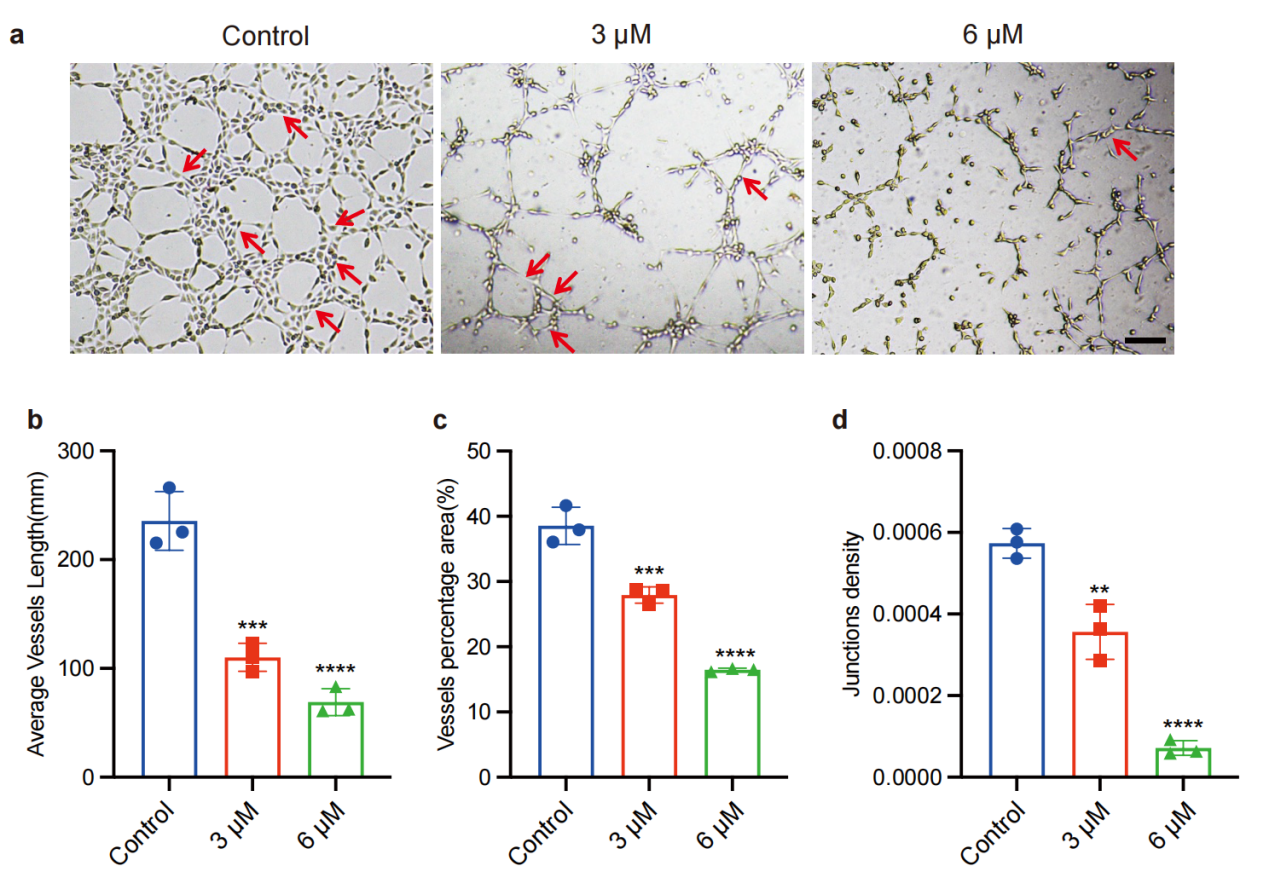
**

**FIGURE S2** GA significantly inhibited the angiogenic ability of HUVECs. (a) Tube forming ability of HUVECs cultured for 4 h after treatment with different concentrations of GA. Scale bar: 20 μm. (b-d) Statistical analysis of vessel length, vessel area and vessel junctions. Data are presented as mean ± SD. n=3.^*^*p* < 0.05, ^**^*p* < 0.01, ^***^*p* < 0.001, and ^****^*p* < 0.0001 when compared with control group.


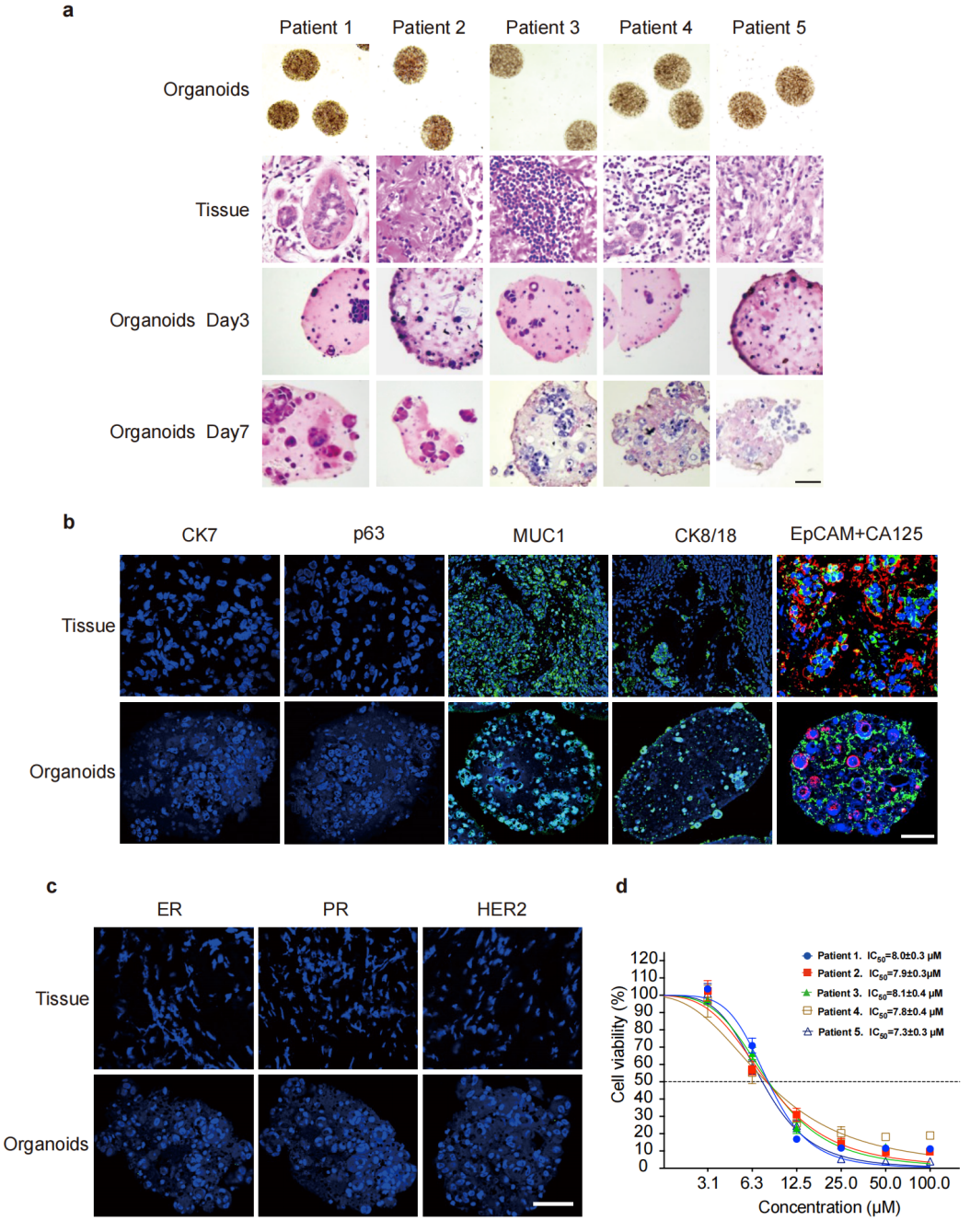


**FIGURE S3** Constructing TNBC organoids to identify the anti-tumor effects of GA. (a) Bright image and HE staining of TNBC patient tissues and organoids. Scale bar: 100 μm. (b) Immunofluorescence staining of CK7, p63, MUC1, CK8/18, EpCAM (Green) and CA125 (Red) in patient tissues and organoids. Scale bar: 50 μm. (c) Immunofluorescence staining of ER, PR and HER2 in TNBC patient tissues and organoids. Scale bar: 50 μm. (d) The identification of anti-TNBC effects of GA by using TNBC organoids.

**
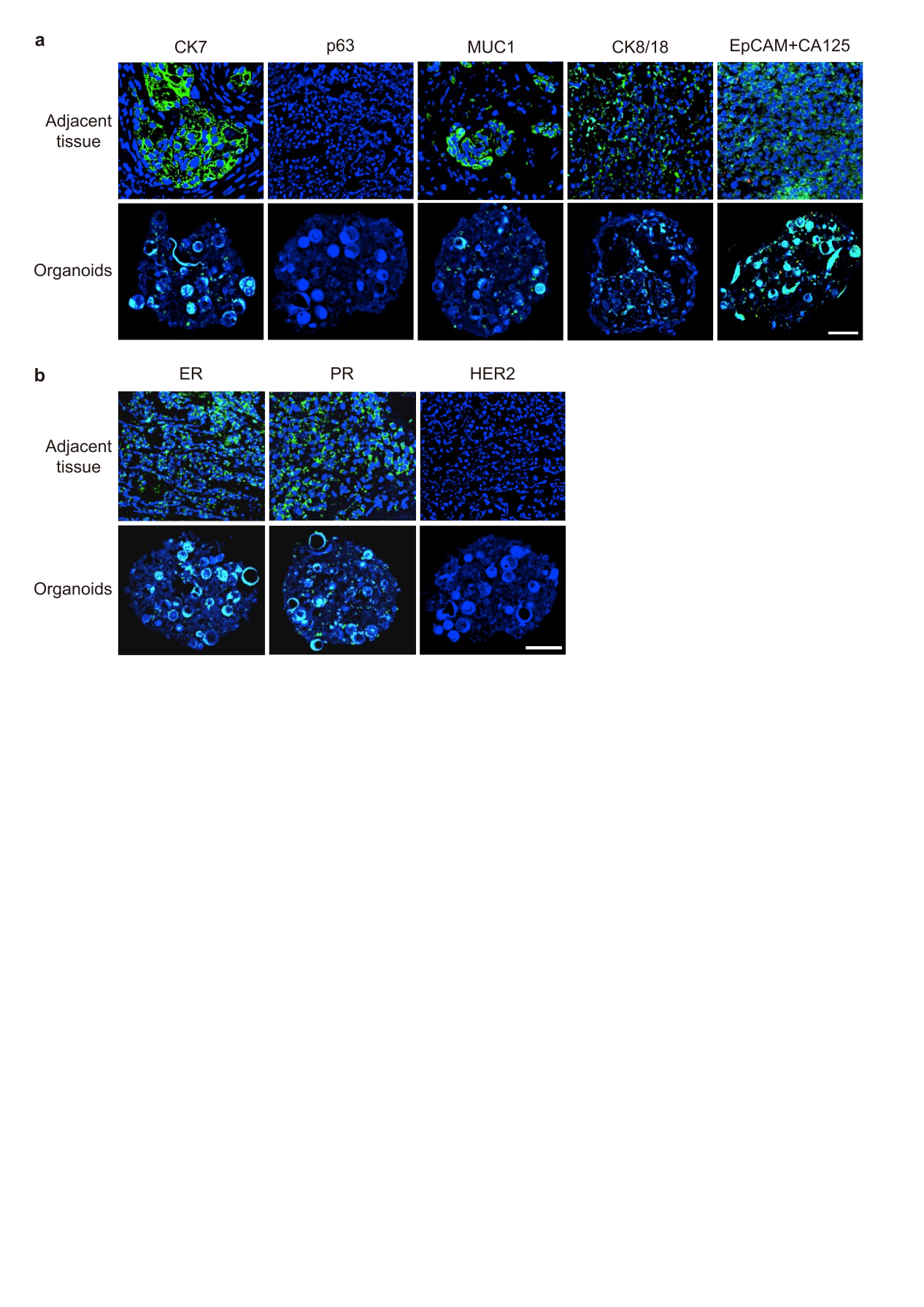
**

**FIGURE S4** Constructing TNBC adjacent tissue organoids. (a) Immunofluorescence staining of CK7, p63, MUC1, CK8/18, EpCAM (Green) and CA125 (Red) in patient adjacent tissues and related organoids. Scale bar: 50 μm. (b) Immunofluorescence staining of ER, PR and HER2 in TNBC patient adjacent tissues and related organoids. Scale bar: 50 μm.

**
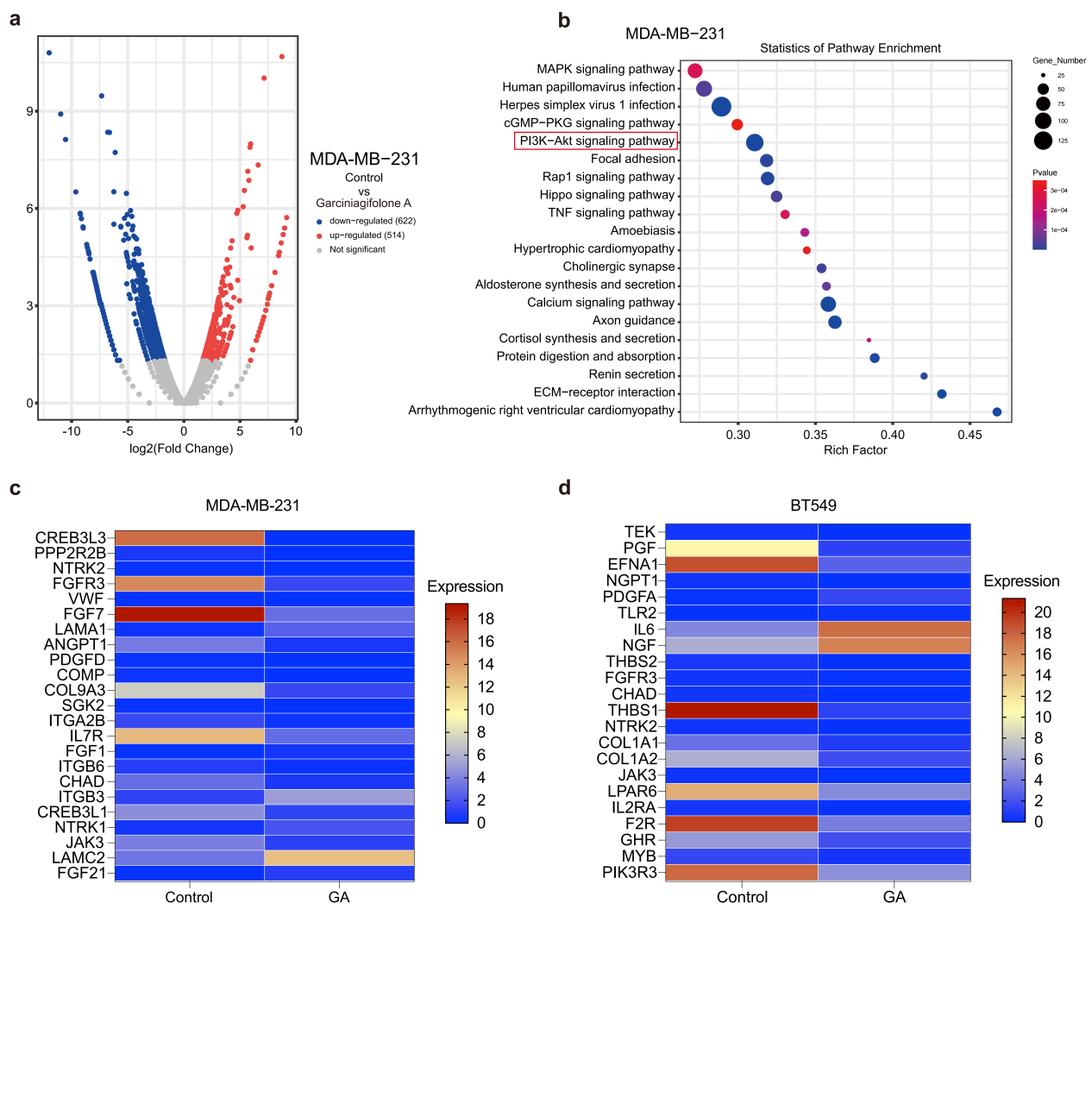
**

**FIGURE S5** The volcano plot and KEGG analysis of MDA-MB-231 cells, and the heatmap of key differentially expressed genes in the PI3K/AKT/mTOR pathway. (a) The volcano plot displayed the differentially expressed genes (DEGs) between the control group and the GA treated group. (b) KEGG data analysis revealed that the PI3K/AKT/mTOR signaling pathway was significantly inhibited after GA treatment compared with the control group. (c) The heatmap of key differentially expressed genes in the PI3K/AKT/mTOR pathway of MDA-MB-231 cells. (d) The heatmap of key differentially expressed genes in the PI3K/AKT/mTOR pathway of BT549 cells.


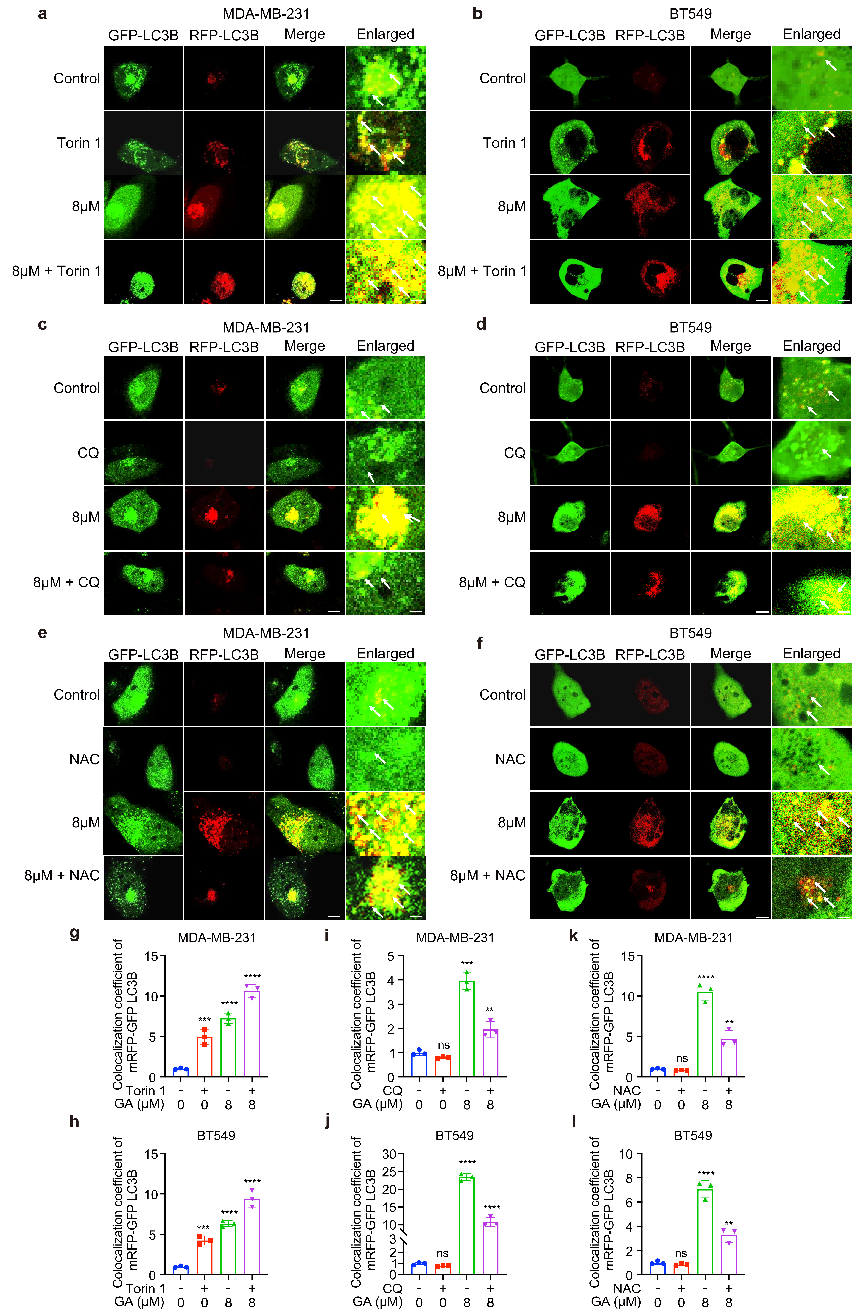


**FIGURE S6** Confocal image and related statistical data of MDA-MB-231 and BT549 cells transfected with mRFP-GFP-LC3B plasmid. Scale bar: 25 μm and 5 μm in normal and enlarged, respectively. Arrow: the colocalization of GFP-LC3B and RFP-LC3B. Data are presented as mean ± SD. n=3.^*^*p* < 0.05, ^**^*p* < 0.01, ^***^*p* < 0.001, and ^****^*p* < 0.0001 when compared with control group.


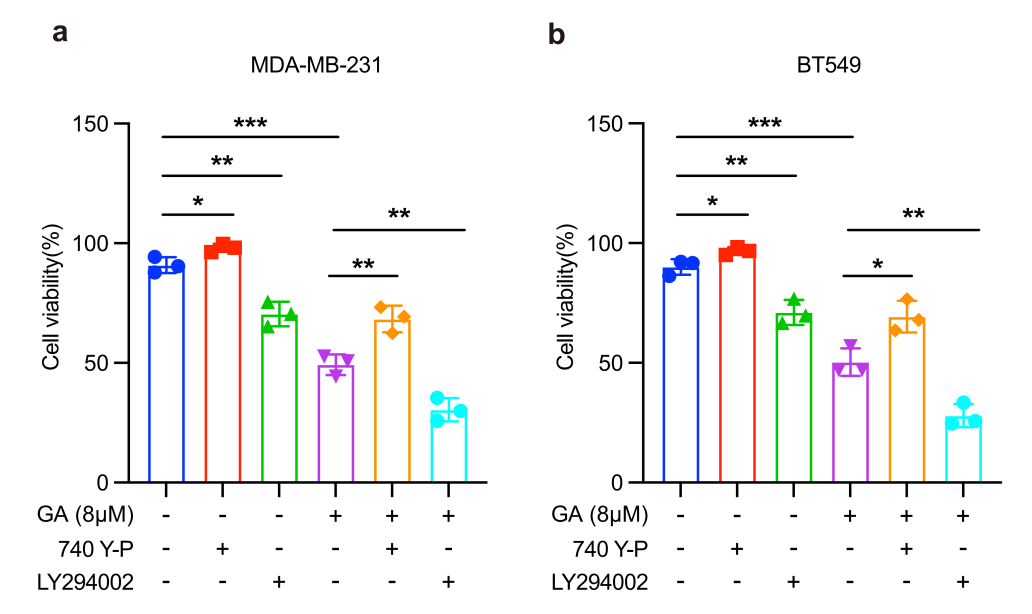


**FIGURE S7** GA induced TNBC cell death via blocking PI3K/AKT/mTOR pathway. (a, b) The viability of MDA-MB-231 and BT549 cells were tested by CCK8 assay. Data are presented as mean ± SD. n=3. ^*^*p* < 0.05, ^**^*p* < 0.01, ^***^*p* < 0.001, and ^****^*p* < 0.0001 when compared with control group.

**
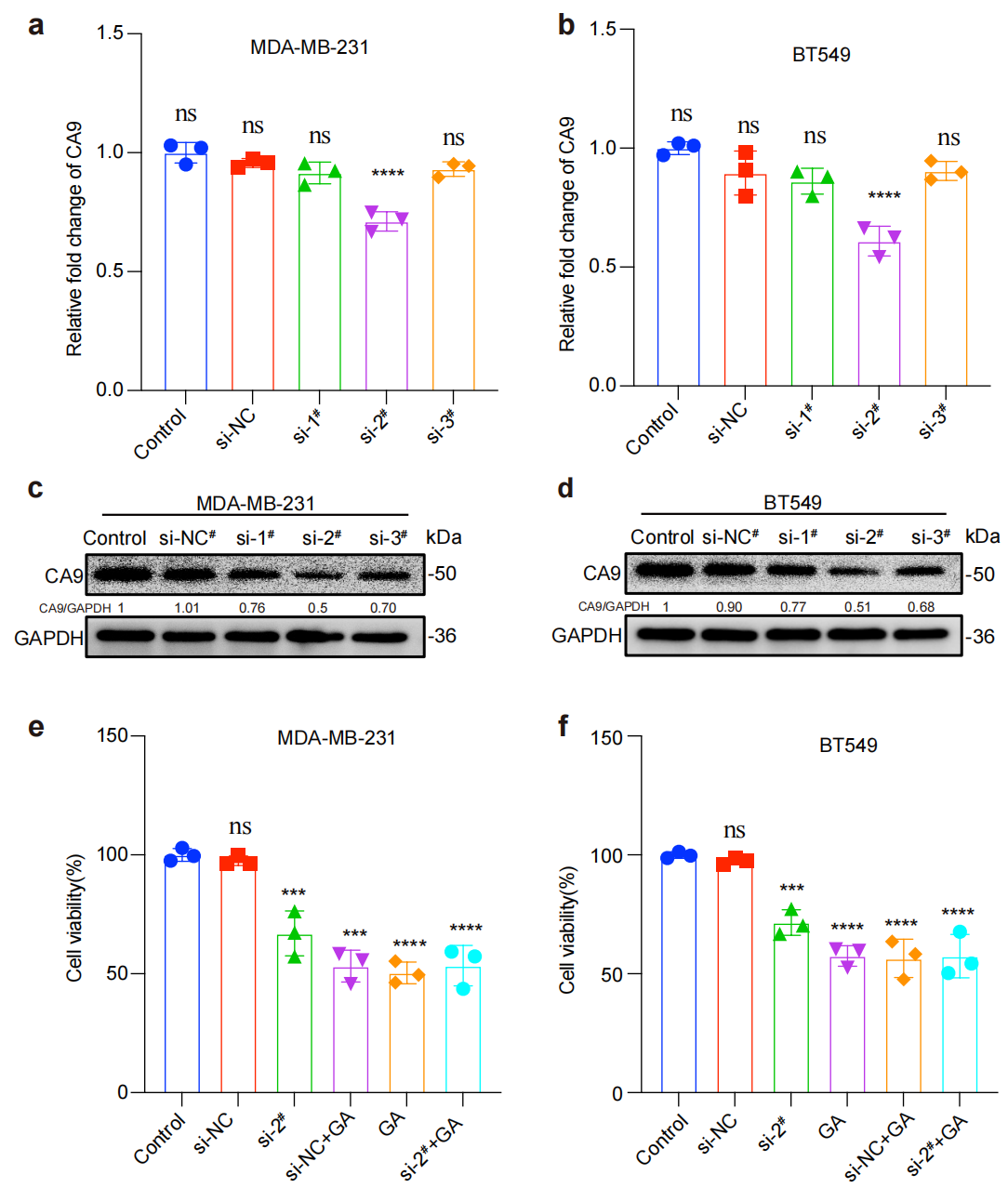
**

**FIGURE S8** GA suppressed CA9 expression in TNBC cells. (a,b) Levels of CA9 mRNA by qRT-PCR in MDA-MB-231 and BT549 cells. (c,d) CA9 protein levels in siRNA-CA9 (MDA-MB-231 and BT549 cells) by Western blotting. (e,f) MDA-MB-231 and BT549 cells viability by CCK8 assay (GA=8 μM). Data are presented as mean ± SD. n=3.^*^*p* < 0.05, ^**^*p* < 0.01, ^***^*p* < 0.001, and ^****^*p* < 0.0001 when compared with control group.
